# Searching for the roots of the first free African American community

**DOI:** 10.1038/s41598-020-77608-8

**Published:** 2020-11-26

**Authors:** Beatriz Martínez, Filipa Simão, Verónica Gomes, Masinda Nguidi, Antonio Amorim, Elizeu F. Carvalho, Javier Marrugo, Leonor Gusmão

**Affiliations:** 1 Molecular Genetics Laboratory, Institute for Immunological Research, University of Cartagena, Cartagena, 36-100 Colombia; 2 DNA Diagnostic Laboratory (LDD), State University of Rio de Janeiro (UERJ), 20550-900 Rio de Janeiro, Brazil; 3 IPATIMUP/i3S, Instituto de Investigação e Inovação em Saúde, Universidade do Porto, 4200-135 Porto, Portugal; 4 Faculty of Sciences, University of Porto (FCUP), 4169-007 Porto, Portugal

**Keywords:** Genetics, Genetic markers, Haplotypes, Population genetics

## Abstract

San Basilio de Palenque is an Afro-descendant community near Cartagena, Colombia, founded in the sixteenth century. The recognition of the historical and cultural importance of Palenque has promoted several studies, namely concerning the African roots of its first inhabitants. To deepen the knowledge of the origin and diversity of the Palenque parental lineages, we analysed a sample of 81 individuals for the entire mtDNA Control Region as well as 92 individuals for 27 Y-STRs and 95 for 51 Y-SNPs. The results confirmed the strong isolation of the Palenque, with some degree of influx of Native American maternal lineages, and a European admixture exclusively mediated by men. Due to the high genetic drift observed, a pairwise *F*_*ST*_ analysis with available data on African populations proved to be inadequate for determining population affinities. In contrast, when a phylogenetic approach was used, it was possible to infer the phylogeographic origin of some lineages in Palenque. Contradicting previous studies indicating a single African origin, our results evidence parental genetic contributions from widely different African regions.

## Introduction

San Basilio de Palenque is a small town near Cartagena, Colombia, founded by runaway slaves (Supplementary Fig. S1). At the end of the sixteenth century, African slaves started to escape from the coastal city of Cartagena to take refuge in the nearby region of Montes de María, establishing, the foundations of the town of San Basilio de Palenque (hereafter referred to as Palenque). Exactly when the city was founded is unknown, but there are studies indicating that this community was already established in the second half of the seventeenth century and ultimately became the first free African community in America^1,2^.

Due to its strategic position located on the north coast of Colombia, Cartagena city was the centre of the Spanish slave trade and one of the main South American ports of arrival for slaves brought from different regions of Africa. The paucity of historical records makes it difficult to establish the exact place of departure of the slaves from Africa. Nonetheless, it is thought that until the early seventeenth century, the Africans arriving in Cartagena would have left from the region of Upper Guinea. Later, the Congo and Angola, together with Upper Guinea, would have been the major regions from which slaves were taken to Spanish America. At the end of the eighteenth century/beginning of the nineteenth century, slaves would have come from several regions, from Senegambia to Mozambique^3,4^.

The village of Palenque is currently inhabited by approximately 4000 Afro-descendants who maintained a cultural and ethnic identity for more than 3 centuries, with high endogamy and little influence from neighbouring communities^5^. Palenque preserves the ethnic conscience and cultural traits of African roots such as the social organisation, complex funeral rituals, and traditional medical practices, among others^6^. Moreover, it is the only African American population speaking a Creole language with a Spanish lexical base^5,7^. Due to these characteristics, Palenque was declared by UNESCO as a Masterpiece of the Oral and Intangible Heritage of Humanity in 2005. During the last decade, the recognition of the historical and cultural importance of Palenque has promoted several studies with the aim of reviving its history and searching for the African roots of its first inhabitants.

Given the high diversity of slaves arriving in Cartagena, one might suppose that several ethnic groups would be behind the foundation of Palenque. However, this theory has been questioned by linguistic and anthropological evidence, pointing to the region between Congo and Angola (the ancient Kingdom of Kongo) as the origin of the first habitants of Palenque^2,3^, with an almost exclusive contribution from a single Bantu ethnic group, the Bakongo, speakers of Kikongo^5^.

However, cultural and genetic features do not always come together, and few genetic studies have been performed to confirm a single geographic source of the founders of Palenque. The first genetic studies carried out with human leukocyte antigen (HLA) markers revealed limited Native American and European gene flow, as well as close genetic distances with African populations, especially from western Africa^8,9^. Nevertheless, a higher than expected European input (38%) was detected when studying the pool of paternal lineages in Palenque^10^. Due to the strong isolation of Palenque, paternal European admixture most likely occurred before its foundation^10^. The results based on autosomal ancestry informative markers showed approximately 10% European ancestry, supporting a sex biased European influx^11^.

More recently, Ansari-Pour et al.^12^ investigated uniparental markers from the Y chromosome and mitochondrial DNA (mtDNA). According to these authors, the Yombe from the Republic of the Congo is the most likely group from which the original male settlers of Palenque came.

Taking together all genetic evidence available, it is safe to assume that Palenque has preserved a high African ancestry and that the European background was essentially mediated by males. Nevertheless, the information available is not enough to clearly assign the continental/regional origin of all parental lineages that are present in Palenque. Concerning mtDNA, the results from Hypervariable segment I (HVSI) did not allow us to determine the continental origin in more than 25% of the studied haplotypes^12^.

For the Y chromosome, the available information is also fragmentary. Data available in Noguera et al.^10^, for a small number of samples, do not allow us to evaluate founder effects, given the lack of information on Y chromosome specific short tandem repeats (Y-STRs). Based on the results from Ansari-Pour et al.^12^, it is not possible to quantify non-African influx due to the low resolution of lineages outside haplogroup E1b1a-M2.

Aiming to obtain a deeper knowledge of the diversity and origin of the Palenque parental lineages, in this study, we analysed the entire mtDNA control region (CR), as well as Y chromosome-specific markers. For both maternally and paternally inherited gene pools, we intended to determine the degree of isolation from Native American and European influx. Based on comparisons with data from African populations, we also intended to contrast the hypothesis of a single against a multiple African origin.

## Materials and methods

A total of 95 male children (aged between 5 and 18) were selected for this study. The volunteers identified themselves as having Palenque descent for at least 3 generations (all parents and grandparents born in Palenque). The samples were collected under written informed consent from the guardians of the participants included in the study. The project and informed consent were approved by Act No. 40 of the ethical committee of the University of Cartagena, Colombia; and the ethical principles of the 2000 Helsinki Declaration of the World Medical Association (http://www.uma.net/e/policy/b3.htm) were followed. Based on genealogical information, only unrelated individuals (not sharing grandparents) for at least three generations were selected. A total of 48 children were recruited at the Benkos Biojó Rural school in Palenque (PR), and 47 children resided in the urban area of Cartagena city (PU). The children from PU were recruited in schools participating in an ethno-education program created to preserve aspects of Palenque culture such as dance, music, religion, and especially the *Palenquero* language^7^.

DNA was extracted from blood samples using a standard salting-out protocol.

The 95 samples were genotyped for 51 Y chromosome-specific single nucleotide polymorphisms (Y-SNPs) using previously described methods (see details in Supplementary Fig. S2). Ninety-two samples were genotyped for the 27 Y-STR loci included in the Yfiler™ Plus kit, following the manufacturer’s protocol (Thermo Fisher Scientific, Waltham, MA, USA). Amplified fragments were separated and detected on a 3500 XL Genetic Analyzer and genotyped using Gene Mapper IDX v.4.0 (Thermo Fisher Scientific).

A subgroup of 81 samples was sequenced for the full control region of mtDNA. The fragments between positions 16024 and 576 were amplified, sequenced, and detected as previously described in Simão et al*.*^13^ using the primers listed in Supplementary Table S1. Haplotypes were classified using SeqScape v2.7 software (Thermo Fisher Scientific). Haplogroups were assigned using both EMPOP database v4/R12^14^ and Haplogrep tool^15^ and confirmed in Phylotree^16^. Data were submitted to the EMPOP database for quality control checks and are available for research purposes under the accession number EMP00749. Mitochondrial DNA sequences were deposited in GenBank: PopSet 1782793150 (https://www.ncbi.nlm.nih.gov/popset/?term=1782793150), accession numbers: MK930265–MK930345.

Haplogroup frequencies were calculated by direct counting. Haplotype and haplogroup diversities, pairwise genetic distances and non-differentiation probabilities were calculated using Arlequin ver. 3.5.1.2 software^17^. Pairwise genetic distances were visualised by multidimensional scaling (MDS) using the software STATISTICA ver.8.0 (www.statsoft.com). Phylogenetic networks were constructed using Network v10.1.0.0 software (http://www.fluxusengineering.com). The number of Y-STRs used to construct the networks depended on the common set available to maximise the representation of African populations for each haplogroup. In most cases, we used a set of 11 loci, namely, DYS389I, DYS389II, DYS19, DYS390, DYS438, DYS392, DYS437, DYS385a/b, DYS393, and DYS439. However, for clades Y-MRCA* (xM13, SRY10831.1) and E1b1b-M35, DYS391 was also included, since it was genotyped in most studies reporting samples from these haplogroups.

## Results

The mtDNA and Y chromosome haplotypes found in this study are described in Supplementary Tables S2 and S3, along with the corresponding haplogroup classifications. Differences in EMPOP and Haplogrep classifications were observed in only one sample (PU063), which was classified as L2a1 + 16189 + (16192) by Haplogrep and L2a1 by EMPOP. The classification from EMPOP is supported by Phylotree, since PU063 lacks 16189C and has a heteroplasmy at position 16192.

The two subsamples from Palenque (PR and PU) were compared by means of *F*_*ST*_ genetic distances and corresponding nondifferentiation probabilities (after 10100 permutations). Regarding mtDNA haplotypes, no statistically significant difference was found (*F*_*ST*_ = − 0.018; *P* = 0.9855 ± 0.001). For the Y chromosome, the PR subsample showed a lower proportion of African Y-SNP haplogroups than the PU (54.2% and 68.1%, respectively). However, differences between subsamples were not statistically significant for Y-STR haplotype distributions (*F*_*ST*_ = 0.0041; *P* = 0.1119 ± 0.0014) or for Y-SNP haplogroups (*F*_*ST*_ = 0.0163; *P* = 0.0792 ± 0.0012). Based on these results, samples were pooled for the remaining analysis.

### The genetic diversity of San Basilio de Palenque

Regarding mtDNA, high haplogroup diversity was found (0.8895 ± 0.0193) for a total of 22 different haplogroups present in our sample. This high haplogroup diversity is, however, associated with a low diversity of haplotypes (0.9225 ± 0.0162). A large proportion of haplotypes were shared inside haplogroups, and only 33 different haplotypes were present in the studied sample. A wide separation of African haplogroups carrying few haplotypes is illustrated in a network (Supplementary Fig. S3). The great majority of the samples (91%) belong to the African macro haplogroup L (Table 1). The remaining 9% belong to Native American haplogroups (A2, A2af1a1, A2al, B2d and C1c3). No European maternal lineages were observed.

**Table 1:** Frequencies of the mtDNA and Y chromosome haplogroups detected in a population sample from Palenque.

mtDNA			Y-SNPs		
*Haplogroup*	*n*	%	*Haplogroup*	*n*	%
L0a1a + 200	1	1.23	Y-MRCA*(xM13, SRY10831.1)	3	3.16
L1b1a + 189	10	12.35	B2a-M150* (xM109)	3	3.16
L1b1a1′4	7	8.64	E1a-M33	2	2.11
L1b1a18	1	1.23	E1b1a-M191	7	7.37
L1b1a7a	1	1.23	E1b1a-M2* (xM154, M191)	36	37.89
L1c3	1	1.23	E1b1b-M35* (xM78, M81, M123, V6, M293)	3	3.16
L1c3a	2	2.47	R1b-V88	4	4.21
L1c3a1b	4	4.94	**AFRICAN**		**61.05**
L2a1	2	2.47	E1b1b-M81	2	2.11
L2a1 + 143 + @16309	1	1.23	E1b1b-M123	8	8.42
L2a1 + 16189 + (16192)	20	24.69	G-M201	1	1.05
L2a1c3b2	1	1.23	I2-M26	1	1.05
L2b1a	1	1.23	J2-M172	1	1.05
L2d + 16129	1	1.23	R1a-SRY1831.2	9	9.47
L3d1a1a	11	13.58	R1b-M529	2	2.11
L3e1d	9	11.11	R1b-S116* (xU152, M529, M153, M167)	9	9.47
L3f1b + 16292	1	1.23	R1b-U152	1	1.05
**AFRICAN**		**91.36**	**EUROPEAN**		**35.79**
A2 + (64)	1	1.23	Q1a2-M3* (xM19, M194, M199)	3	3.16
A2af1a1	2	2.47	**NATIVE AMERICAN**		**3.16**
A2al	1	1.23			
B2d	1	1.23			
C1c3	2	2.47			
**NATIVE AMERICAN**		**8.64**			

Note: The presence of E1b1b-M81 in Palenque is interpreted in this study as the result of European admixture, although it cannot be ruled out that it came from North Africa via western Africa. Although an African origin was considered to be more likely for the E1b1b-M35* (xM78, M81, M123, V6, M293) lineage in Palenque, we cannot exclude the hypothesis that it came from Europe, since rare E1b1b-M35 subclades were reported in European populations.

A high diversity was also found for Y-SNP haplogroups (0.8185 ± 0.0033), with 17 different haplogroups being observed (Table 1). Similar to mtDNA, low Y-STR haplotype diversity was observed (0.9881 ± 0.0038), with many shared haplotypes inside haplogroups (Supplementary Fig. S4). In the whole sample, an African origin can be attributed to 61% of the Y-haplogroups, 36% represent European admixture, and three samples (3%) belong to a Native American haplogroup (Table 1).

### The African origins of San Basilio de Palenque lineages

To investigate the origins of the African lineages in Palenque, we selected subsamples of African haplogroups found for both mtDNA and the Y chromosome. These subsamples were compared with populations from different African regions (see Supplementary Tables S4 and S5). A pairwise comparison was performed among all selected populations. Moreover, phylogeographic analyses of the African haplogroups in Palenque were undertaken based on haplogroup frequencies and distributions across the African continent and considering the haplotypic profile of each lineage.

#### ***Population pairwise F***_***ST***_*** analysis***

**Figure 1:**
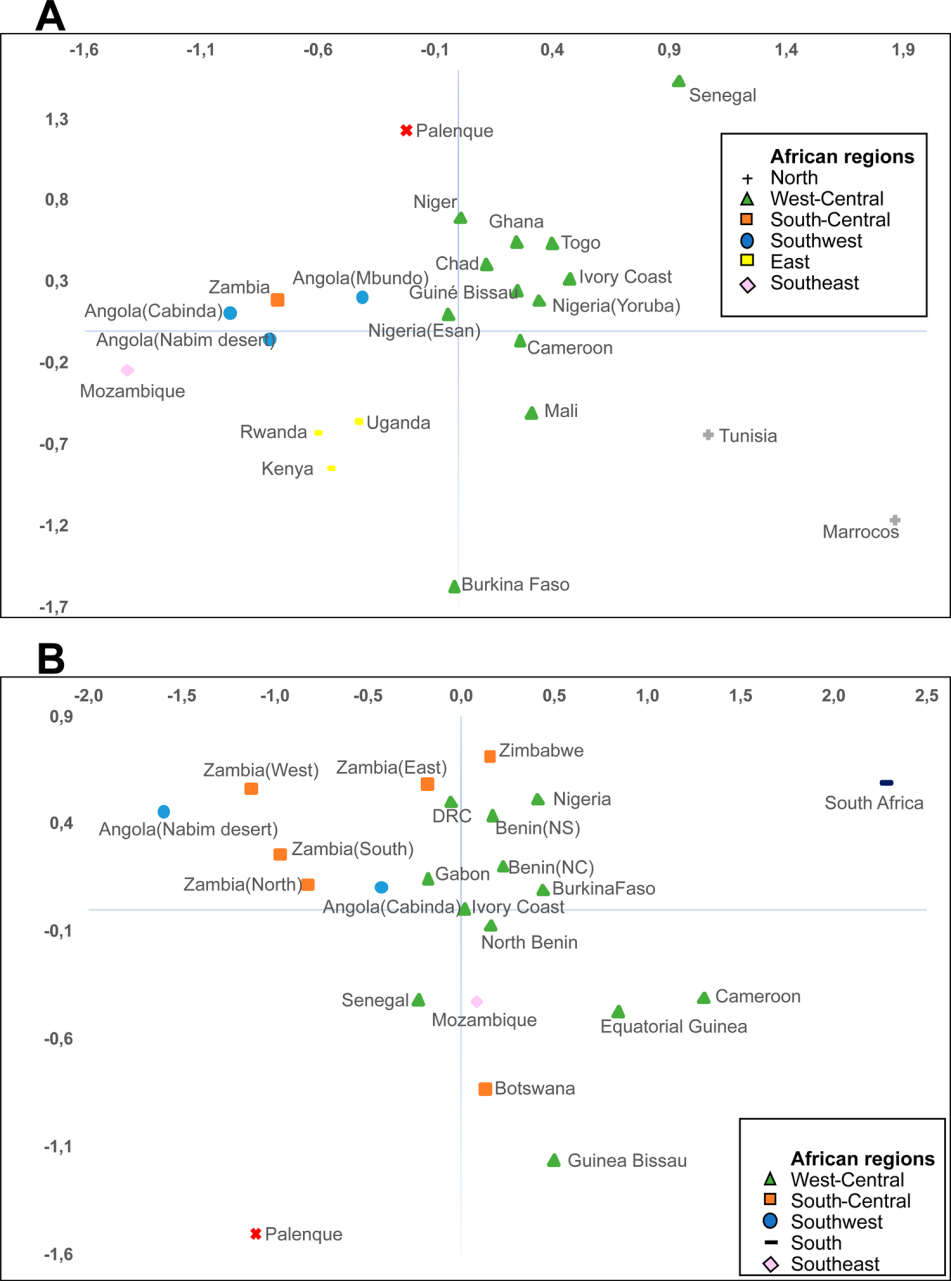
MDS plot of pairwise *F*_*ST*_ genetic distances between the subset of African lineages in Palenque and different African populations, based on (**A**) mtDNA (HVSI and HVSII) haplotypes and (**B**) 10 Y-STRs (DYS389I, DYS389II, DYS19, DYS391, DYS390, DYS438, DYS392, DYS437, DYS393 and DYS439).

For the mtDNA (HVSI and HVSII), *F*_*ST*_ genetic distances were calculated between the subgroup of L-lineages in Palenque and haplotype distributions in African populations (see Supplementary Table S4). Relatively high *F*_*ST*_s and low non differentiation probabilities were observed between Palenque and all African populations used for comparison (see Supplementary Table S6). As depicted in the MDS plot (Fig. 1A), Palenque is far from all populations, although closer to the western ones. A similar picture emerges from the comparative analysis between the Palenque Y-chromosomal African pool and reference populations from Africa (see supplementary Table S5). Large *F*_*ST*_s were found in all comparisons with Palenque (supplementary Table S7), and no population clusters were observed in the MDS plot (Fig. 1B).

#### **Phylogeographic analysis of the African mtDNA lineages**

The African lineages in Palenque fall into four clades, L0, L1, L2 and L3, including 17 different haplogroups. The African origin of each haplotype in Palenque was investigated by comparison with data from African populations (Supplementary Table S4).

The southeast and east regions of Africa were identified as the likely origin for the most ancient L0 lineages (such as L0k and L0d)^18,19^. Previous studies show that L0 reaches the highest frequencies and sub-haplogroup diversities in these areas^18^. In contrast, due to a later northeast migration, more recent L0 subclades such as L0a1a show the highest frequencies in central Africa^19–21^. A network built using data available in the literature for L0a1a + 200 shows that none of the African haplotypes match the haplotype from Palenque (Supplementary Fig. S5). The closest haplotypes were from east (Uganda)^22^ and west populations (Guinea Bissau and Togo)^23,24^. Considering historical records, an origin of this lineage in the west-central region is more likely than in East Africa.

A total of 19 samples from Palenque had haplotypes classified inside L1b (Table 1). Branches inside L1b are spread throughout Africa, although the branches are more frequent in the west-central region^20,25^. Both L1b1a + 189 and L1b1a18 branches were found in several regions throughout Africa, as shown in a network (Supplementary Fig. S6). Due to their broad distribution, it was not possible to point to the origin of these lineages in Palenque. L1b1a7a was not found in the collected population data, and L1b1a1′4 was found in one sample from Guinea Bissau and one from Senegal^23,26^. The two L1b1a1′4 haplotypes in Palenque differ by a single mutation, and the most frequent one is separated from the Guinea Bissau and Senegal haplotypes by one step (Supplementary Fig. S6). A search in EMPOP (empop.online, release v4/R13)^14^ showed the presence of 13 and 25 samples from L1b1a1′4 and L1b1a7a, respectively, most from admixed populations in America. Apart from the presence of L1b1a1′4 in Guinea Bissau, these haplotypes could not be found in other African populations in the EMPOP.

The territory between Cameroon and Angola has been identified as the most likely origin for L1c^27,28^. Additionally, in this region, this haplogroup currently has the highest frequencies. L1c was observed in 7 samples from Palenque, distributed among three sub-haplogroups: one sample is L1c3, one is L1c3a and four are L1c3a1b. Additionally, one haplotype showed a point heteroplasmy at position 16355, necessary for haplogroup classification: a C at position 16355 corresponds to L1c3; a T variant puts the sample in L1c3a1b (Supplementary Table S2). A network was built for haplogroups L1c3, L1c3a and L1c3a1b (Supplementary Fig. S7). Approximately, 94% of the African samples are from south central, southwest, or west-central populations. Only a small number were from the east or southeast. In accordance with previous studies^25,28^, L1c revealed high internal haplotype diversity, preventing a detailed investigation of the origin of the Palenque lineages. Nonetheless, three out of five L1c3a1b samples from Palenque shared a haplotype with a sample from Ghana^29^ (Supplementary Fig. S7). This haplotype is one mutational step apart from two Bantu samples from Angola^30^ and two mutational steps from one sample from Cameroon^18^ (Supplementary Fig. S7). Consequently, an origin of the Palenque lineages in the western region (central and/or south) seems more likely than in the eastern or north regions.

Haplogroup L2, which has been associated with Bantu expansion, has its most likely origin in western Africa. Currently, L2 is spread throughout the continent, presenting high frequencies in central west and southeast Africa^25,31^. Three (L2a, L2b and L2d) of the five main branches from L2 (L2a-L2e) were found in Palenque (Table 1). L2a is the most common and widespread clade in sub-Saharan Africa, and was described with the highest frequencies in Ghana, Sudan and Mozambique^25,31^. Approximately 92% (n = 24) of the L2 found in Palenque was attributed to branches inside L2a. In the networks of L2a1 (Supplementary Fig. S8) and L2a1 + 16189(16192) (Supplementary Fig. S9), it is possible to see a wide geographic dispersion, typical of L2a lineages. In both cases, a star-like distribution emerges from a central cluster of shared haplotypes from several African regions. The two L2a1 samples from Palenque are one and/or two mutational steps from the central cluster. All haplotypes from L2a1 + 16189(16192) are associated with a single founder who shares its sequence with samples from several African regions. The only L2a1 + 143 + 16309 haplotype from Palenque is separated by one position from west, southwest and east Africa haplotypes (Supplementary Fig. S10). A L2a1c3b2 sample from Palenque differed from the only African sample available for this haplogroup^32^ in 5 polymorphic sites.

In summary, inferences concerning the African origin of Palenque haplotypes inside the L2a clade were not possible due to its wide distribution in Africa, as well as because of the uncertainty in subbranche classification associated with rapidly mutating polymorphisms^25,31^.

The L2b and L2d subbranches are less frequent in Africa than L1a^25,27,31^. Both haplogroups originated in western Africa, showing the highest frequencies in west-central and southwest populations. Although L2b1a is characterised by the 146T and 16355T polymorphisms, the L2b1a sample from Palenque lacks the 146T mutation. No samples in the literature were found to have this exact profile. In the network, the haplotype from Palenque differs from the central cluster by two mutational steps (the first one being 146) (Supplementary Fig. S11). Comparably, one sample from Cameroon^24^ also diverges from the central cluster by a polymorphism at position 146.

L2d is the oldest L2 subclade^25^ and shows a sporadic occurrence on the African continent. The L2d + 16129 haplotype from Palenque matches the single L2d + 16129 sample from Angola (Bantu)^30^ (Supplementary Fig. S12). The widespread distribution previously reported for other L branches is once again observed in the L2b and L2d sub-lineages. Nevertheless, a clear southwest origin for the L2d haplotype in Palenque can be demonstrated. Regarding the L2b haplotype in Palenque, data place the most likely origin in the western region (between the Bight of Benin and Angola), while east and southeast origins can possibly be excluded.

L3 is thought to originate in east Africa, the homeland for out-of-Africa migration and diversification, where it reaches the highest frequency^21,25^. Nonetheless, L3 subbranches can also be found in other African regions. Approximately 28% of the L-lineages found in Palenque are distributed across three L3 branches: L3d, L3e and L3f.

The L3d branch is very common in west-central Africa^25^, even though it is also present in other regions such as the east and southeast. Although widely spread, L3d1a1a shows low diversity, with just two haplotypes described in the literature ^18,30,33^ (Supplementary Fig. S13). The eleven L3d1a1a samples from Palenque have the same haplotype. It was nevertheless impossible to infer the exact region of Africa where this haplotype originated, since this haplotype (i) was not found in the African samples, and (ii) differs by one step from a central cluster that includes samples from several regions (Supplementary Fig. S13).

Compared to other L3 subclades, L3e is the most frequent and widely distributed in the African continent. It is especially common in Bantu groups^25^. The L3e1d network shows an almost exclusive distribution in southwest^18,30^ and south-central Africa^34,35^ (Supplementary Fig. S13), indicating a southern origin for this lineage in Palenque. All samples from Palenque share the same haplotype (apart for one variant of a heteroplasmic sample), suggesting a single founder.

Haplogroup L3f is rare in Africa^25^. L3f originated in the eastern region and later expanded to central Africa. This branch is also present in west-central Africa^25^. Although no matching haplotypes were found with the L3f1b + 16365 sample from Palenque, the closest one was from Ghana (Supplementary Fig. S13).

#### **Phylogeographic analysis of the African Y chromosome lineages in Palenque**

The African paternal lineages found in Palenque fall into four different clades: Y-MRCA* (xM13, SRY10831.1), B, E and R, comprising seven haplogroups. The most likely origins of these haplogroups were investigated by comparing the Y-STR haplotype profile of samples from Palenque and African populations (Supplementary Table S5).

In this study, we detected three chromosomes classified as Y-MRCA* (xM13, SRY10831.1), which includes samples from haplogroup A. Haplogroup A is virtually restricted to the African continent, reaching the highest frequencies in Khoisan-speaking populations. It is also frequent in the Nilotic groups from east and northeast Africa^23,36–38^, and it was sporadically observed in the southeast and southwest populations^39,40^. Some lineages inside clade A have geographic specificity. For instance, lineages belonging to haplogroups A1-M31 were described in west Africa^41^, differing from those in the eastern part of the continent that belong to haplogroups A3-M13 or to other lineages inside A3.

To infer the most likely origin of the haplogroup A samples found in Palenque, a network was built, including the Y-STR profiles of two samples from this study (we had no Y-STR information for one sample) and those from 109 haplogroup A samples compiled from the literature (Supplementary Table S5). The network constructed depicts a clear separation of samples belonging to haplogroups A1-M31 (from Guinea Bissau, in west Africa), A3-M13 (including all samples from East Africa, some from northeast Africa, and one sample from Benin and one from Equatorial Guinea) and A3-M28 (including four samples from northeast Africa) (Supplementary Fig. S14). Central African Bantu and Pygmy samples from Ghana and Cameroon, not typed for Y-SNPs inside haplogroup A [Y-MRCA (xA4)], separate into two clusters, not overlapping A1-M31, A3-M28 and A3-M13 clusters. The two samples from Palenque (which share the full Yfiler Plus profile) stand between the A1-M31 and A3-M28 clusters. Although it is difficult to point to an origin of the haplogroup found in Palenque, we can at least exclude that they came from east, southeast, or south African regions. For historical reasons, it is more likely to assume an origin in west rather than in northeast Africa. The closest west African branches to the Palenque haplotypes correspond to samples from Guinea Bissau, belonging to haplogroups A1-M31.

Haplogroup B is present exclusively in sub-Saharan African populations, except for recent migration to other continents. Haplogroup B2b-M112 chromosomes are found at their highest frequency among Pygmies and are also frequent in Khoisan, with some lineages being virtually restricted to these hunter-gatherer groups^42,43^. In contrast, subclade B2a is widely dispersed throughout sub-Saharan Africa^44,45^. The presence of this haplogroup in different African regions was first attributed to dispersion of Bantu speakers. Nevertheless, recent studies showed that haplogroup B2a was already present in Khoisan groups before their contact with Bantu-speaking populations^43,45^. We identified three samples belonging to B2a-M150* in Palenque (5.17% of the African lineages), all lacking the M109-derived allele usually present in southwest, southeast and south African Bantu populations^40,46,47^. Subclade B2a is widely dispersed throughout sub-Saharan Africa^44,45^. The network shows a wide dispersion of the haplotypes (Supplementary Fig. S15), which can be due to the low resolution of some lineages. Aiming at broad population coverage, we have included all possible B2a-M150* (xM109) chromosomes, even those that were typed only for the haplogroup B diagnostic SNP. Only 33 of the 134 African reference samples included in the network were typed for M109: 3 from Eritreia, 25 from Uganda, 4 from Nigeria and 1 from Ghana. The samples from Palenque are positioned outside the two main clusters in a branch rooted in a sample from Gabon. Two samples share their haplotypes with a sample from Cabinda (Angola), and the third sample differs by a single step mutation. The results obtained are thus compatible with a single founder, who could have been brought from the ports of Loango (in modern Gabon) or Cabinda (Angola), in the ancient Kingdom of Kongo.

Clade E is the most common clade in Africa and is also present in European and western Asian populations. In accordance with the Palenque African background, this clade represents 61.05% of the Y-lineages and 82.76% of those assigned to an African origin. The haplogroup E1b1b-M81 is frequent in north Africa, particularly in Berber groups and is also present in Iberia and southern Italy as a northwest African legacy^48,49^. Since no direct geneflow from north Africa has been reported, the presence of this haplogroup in the Palenque population most likely resulted from European admixture. Nonetheless, a northern African origin of the E-M81 in Palenque, via western Africa, cannot be ruled out. A European origin was also attributed to the haplogroup E1b1b-M123 lineages found in Palenque. This haplogroup is absent in Sub-Saharan Africa and frequent in Arabian populations from northeast Africa and the Middle East^50^. This haplogroup also spread in Europe, although at low frequencies. In Iberia, it has been associated with Middle Eastern, North African, and Jewish ancestry. A direct origin in Africa can be attributed to the remaining E-haplogroups in Palenque [E1a-M33, E1b1a-M2* (xM154,M191), E1b1a-M191 and E1b1b-M35* (xM78,M81,M123,V6,M293)], which are not present in European populations and were more likely brought to America during the slave trade.

Apart from E1b1b-M81 and E1b1b-M123 (attributed to European influx), inside the E1b1b clade, we found three samples belonging to E1b1b-M35* (xM78,M81,M123,V6,M293). Although an African origin of this lineage is more likely, we cannot exclude the hypothesis that it came from Europe, as rare E1b1b-M35 subclades (harbouring V1515, V257 or V2009 mutations) were detected in populations in the western Mediterranean region, namely, Portugal, Spain, France and Italy^51^.

E1b1b-M35*(xM78,M81,M123,V6,M293) is present in eastern and southern African populations^37,47,48^. It was not detected in central and southwest regions, namely, Niger, Nigeria, Cameroon, Equatorial Guinea, Gabon and Angola^30,42,46–48,52^, although it was described in west Africa at low frequencies. The E1b1b-M35 network shows many separated branches (Supplementary Fig. S16), some of which are restricted to a single African region. The high diversity and wide dispersal in African populations is explained by the low resolution of the data available and most likely represents diverse E1b1b-M35 sublineages. From the network, we can see that the samples from Palenque (represented by a single haplotype) are positioned in a branch that is rooted in a haplotype shared between a sample from Burkina Faso and Ethiopia. Apart from the samples from Palenque, this branch comprises one sample from Senegal, one from Benin, three from Guinea Bissau and two from Burkina Faso. The results obtained are thus compatible with a single founder who is more likely to have come from a region between Senegambia and the Bright of Benin than from other African regions.

Two samples from haplogroup E1a-M33 were found in Palenque, corresponding to 3.45% of the African chromosomes. This haplogroup is present exclusively in west Africa. The highest frequencies reported were in the region of Mali and Burkina Faso^38,47^, with a gradual decrease in frequency towards the south. It was not detected in eastern and southern populations^23,41,47,53^. A network was built using available information on 77 samples from haplogroups E1a-M33 (Supplementary Fig. S17). The two samples from Palenque are well apart from each other. One is in a branch together with samples from Guinea Bissau. This branch is rooted in a cluster mainly comprising samples from Burkina Faso. The other sample is positioned in the network close to a group of samples from Benin and Nigeria. The results obtained are thus compatible with two different origins in western Africa. One of the Y chromosomes is more likely to have come from the region of Upper Guinea. The origin of the other Y chromosome is more likely to be somewhere along the Bight of Benin.

The haplogroup E1b1a-M2 (and its sub-lineages) is widely spread in Africa and highly prevalent in all Bantu sub-Saharan populations, with frequencies above 80% in most populations^39,40,46,47^. In Palenque, 45.26% of the samples (74.14% of the African samples) belong to the haplogroup E1b1a-M2*(xM154,M191) and to its sub-clade E1b1a-M191. The extremely high number and diversity of samples that can be included in these haplogroups did not allow us to resolve the reticulation obtained in the networks (data not shown). Therefore, for these two haplogroups, we carried out a match analysis between the haplotypes found in Palenque and those reported in African populations from the same haplogroups.

A total of 2204 samples, selected from haplogroup E1b1a-M2 (excluding those carrying M154- or M191-derived alleles), were compared with the 35 E1b1a-M2*(xM154,M191) samples from Palenque (Supplementary Table S8). These 35 samples resulted in 9 different clusters, including identical and neighbouring haplotypes (Supplementary Fig. S4). No matches were found for five out of the 9 clusters (Supplementary Table S8); therefore, it was not possible to infer their origin. For two clusters, matching haplotypes were only found in Benin, with seven and one samples, respectively (Supplementary Table S8), placing the most likely origin of these lineages in the region of the Bight of Benin. For the two remaining clusters, identical haplotypes were found mainly in populations south of Cameroon; therefore, these lineages most likely came from the African west coast between Loango and Angola.

From E1b1a-M191, we selected 934 samples from 20 countries in sub-Saharan Africa to be compared with the seven E1b1a-M191 samples found in Palenque (Supplementary Table S9). Three of these samples shared the same haplotype (Supplementary Fig. S4). Exact matches were found for only two of the five different haplotypes. For one haplotype, a match was found in Gabon. The second haplotype has four matches, one in Gabon, two in Angola and one in Zambia, placing the most likely origin of these lineages in a region south of Cameroon, possible between Loango and Angola.

The haplogroup R1b-V88 was found in 6.15% of the African lineages in Palenque. Clade R originated outside Africa and has high frequencies in European populations. However, except for rare sub-lineages, the R1b-V88 sub-clade is essentially restricted to the African continent, with high frequencies in the central Sahel populations^54^. In sub-Saharan Africa, this haplogroup has the highest frequencies in the northern Cameroon Chadic groups^55^. In western Africa, this haplogroup was also found in Gabon and Equatorial Guinea and is absent in the southern populations^42,53,55^. Based on its geographic distribution and diversity levels in different regions, haplogroup R1b-V88 has been associated with the trans-Saharan spread of proto-Chadic populations during the early mid-Holocene^54,55^. A network was built using available information on 56 African samples from haplogroup R after excluding samples that were assigned to sublineages outside the R1b-V88 branch and believed to result from recent European migration (Supplementary Fig. S18). All four samples from Palenque share a Y-STR haplotype, which is located close to four out of the seven R1b-V88 chromosomes found in the Punu people from Gabon (two are separated by a single-step mutation and two by two-steps). In the same cluster is also one sample from Equatorial Guinea and another from Benin (both are two steps apart from the Palenque samples). The results obtained are compatible with a single founder from western Africa. Close haplotypes were found spread along the Bights of Benin and Biafra and along the Loango coast. The closest genetic proximity was found with two samples from Punu-speakers, putting their most likely origin in the current region of Gabon.

## Discussion

### **Ancestry profile of Palenque**

Although previous studies have been undertaken to establish maternal and paternal ancestral contributions to the current population of Palenque, only rough estimates are available due to poor data resolution. In the present study, we analysed numerous Y chromosome-specific markers and extended the mtDNA analysis to the whole CR, enabling a clear assignment of the continental origin of the lineages in Palenque.

A high African maternal ancestry was found, in accordance with the report by Ansari-Pour et al.^12^. Nevertheless, it was not possible to perform a straightforward comparison between the two studies, since previous data included only HVSI information, and many samples could not be assigned to a specific haplogroup, namely, inside some African and Native American branches. Even so, a comparison between HVSI haplotypes showed no statistically significant differences between the two studies (*F*_*ST*_ = 0.0062; *P* = 0.1278 ± 0.004).

In contrast with mtDNA, a high influx of European males was observed, and only three out of the 95 Y chromosomes have a Native American origin. Although a previous study reported an even higher European male lineage input (38.5%), a European origin was attributed to all R1b lineages^10^. However, in the present study, we found samples in Palenque that belong to an African R1b subhaplogroup, characterised by the V88-derived allele, not investigated in the previous study of Noguera et al.^10^. Due to different resolutions in the haplogroup definition, we could not make a meaningful comparison between our data and that from Ansari-Pour et al.^12^. In comparison with this study, Ansari-Pour et al.^12^ performed a more detailed characterisation of the E1b1a-M2 sublineages (including the U175, U181 and U290 markers) but did not distinguish between African, European or Native American haplogroups inside BF-M213* (xM9) and QR-92R7* (xSRY10831.2).

The overall results show that non-African admixture was mainly mediated by European males, responsible for the introduction of at least 9 different haplogroups present in our sample, and no admixture with European women was detected. Native American influx was higher for the maternal than for the paternal lineages. The three Native American Y chromosomes detected could be explained by a single entry, since their haplotypes differ only at DYF387S1, which is known to have a higher than average Y-STR mutation rate (> 1 × 10^−2^)^56^.

### **African roots of Palenque**

One of the most debated subjects regarding the history of Palenque has been the origin of its inhabitants and, hence, the question of whether their ancestors had a single or a multiple origin in Africa. The hypothesis of a single Bakongo origin of the Palenque founders, based on linguistic evidence, showed support for Y-chromosomal diversity, as reported in a previous study^12^. In the study from Ansari-Pour et al.^12^, a comparative analysis between Palenque and populations representing different sub-Saharan African groups showed a close *F*_*ST*_ genetic distance with the Yombe group from the Republic of the Congo for the Y chromosome. However, regarding mtDNA lineages, no evidence was found concerning maternal African roots^12^. In contrast, our results based on pairwise genetic distances did not allow us to place the origin of the Y-haplotypes from Palenque in a specific African region. An AMOVA performed between Palenque in one group and all African populations in a second group revealed a higher variation between Palenque (subsample of African lineages) and the African group (1.76%) than among populations from different African regions (0.88%). The large *F*_*ST*_ between the African substrate of Palenque and all African populations can only be explained by genetic drift, which is in accordance with the low haplotype diversity within Y chromosome haplogroups. Regarding mtDNA lineages in Palenque, similar to the observations by Ansari-Pour et al.^12^, it was not possible to establish maternal African roots based on pairwise genetic distance analyses.

Overall, diversity and genetic distance results from both mtDNA and Y chromosome data underline the importance of genetic drift in the separation of Palenque from its homeland population(s). Therefore, the analysis of genetic distances based on allele frequency distributions is not the best strategy to look for affinities in Africa. In this situation, *F*_*ST*_ genetic distance or principal component analysis will reflect random drift rather than distant genetic affinities. A phylogeographic approach of each of the lineages present in the Palenque is therefore more suitable for the search for their origins in different African regions.

**Figure 2:**
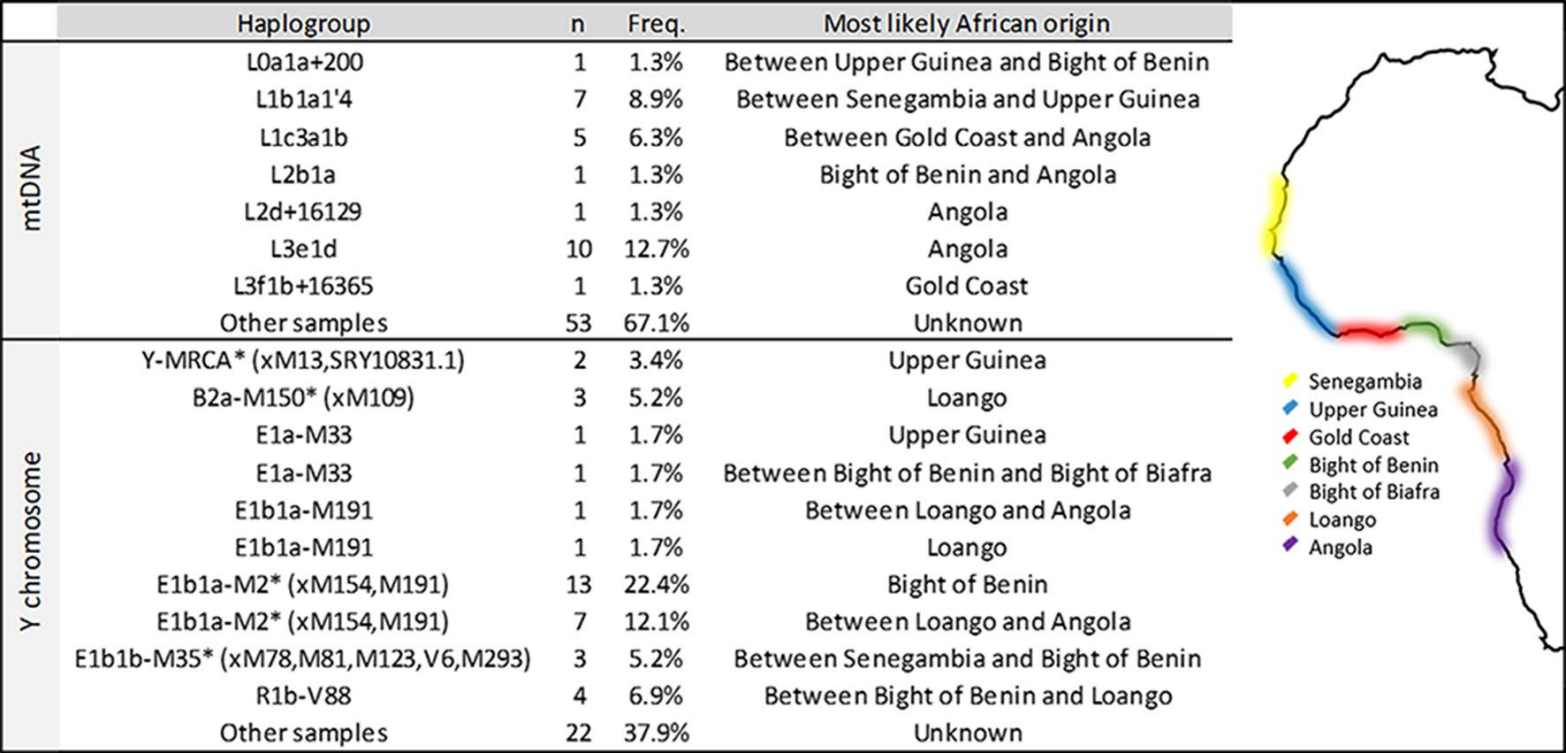
Most likely African origin of the mtDNA and Y chromosome lineages detected in Palenque.

Among the 79 samples from Palenque, at least 17 potential mtDNA founders could be identified (Supplementary Fig. S3). The comparison with data from Africa allowed us to place the most likely origin of seven of these founders (Fig. 2). For almost 70% of the African mtDNA sequences in Palenque, it was not possible to infer the origin. Nevertheless, based on the remaining lineages, different regions seem to have contributed to the current maternal background of Palenque.

Among the 58 samples from Palenque attributed to African Y-haplogroups, at least 20 potential founders could be identified based on haplotype diversity. The comparison with data from Africa allowed placing the most likely origin of 12 out of the 20 potential founders. The results pointed to multiple origins in western Africa in a territory extending from Upper Guinea to Angola (Fig. 2). Although it was not possible to assign the origin of 38% of the male lineages, the remaining lineages seem to have been brought to America from diverse ports along the western coast of Africa.

The detection of lineages originating mainly from the west coast of Africa, as well as their variety of origins, reflects the existing information on the arrival of slave vessels in Cartagena. According to the Slave Voyages database (https://www.slavevoyages.org/), ships from a wide variety of ports in west Africa, from Senegambia to Angola, arrived in Cartagena during the slave trade.

Although the mtDNA for the full control region was analysed in this work, in comparative analyses, we could use only HVSI and HVSII information to comprise the most relevant African regions. For the same reason, only a small set of all genotyped Y-STRs could be used. Moreover, there is little overlap among publications concerning the Y-SNPs used for haplogroup determination. While affinities were found between Palenque and some African regions, it was not possible to pinpoint the origin for many lineages, and some were traced to a vast region, preventing the estimation of the exact proportion in which different African regions contributed to Palenque. The phylogeographic approach used is strongly influenced by the knowledge of the genetic diversity of the original populations, being conditioned by the existence of data with high resolution. However, the gene pool of sub-Saharan African populations is still understudied, and some relevant areas in the history of the trans-Atlantic slave trade have been poorly investigated thus far.

In conclusion, the results from this study showed that the African lineages in Palenque resulted from a restricted number of founders from multiple geographic origins. The results do not contradict the important influence of the Kikongo speakers in the early Palenque, as largely documented and patent in the *Palenquero* language. Nevertheless, this results show that there are still lacunae in the history of the Palenque people, who seem to carry in their genes other roots beyond the Kikongo. The results are compatible with the presence of several African substrates in the early inhabitants of Palenque, although one has been dominant in terms of culture and language. Concerning the European paternal legacy, it remains to be investigated at which point in the history of the Palenque these lineages would have been introduced.

## Supplementary information

Supplementary Information.

Supplementary Information.
