# Supplementary material for: Searching for the roots of the first free African American community
Source: Sci Rep. 2020 Nov 26;10:20634. doi: 10.1038/s41598-020-77608-8 (PMC7691995; doi:10.1038/s41598-020-77608-8)
Supplement: Supplementary file 1 — Supplementary Information. [file 41598_2020_77608_MOESM1_ESM.pdf]

Scientific Reports (2020)

SUPPLEMENTARY FIGURES

Searching for the roots of the first free African American community

Beatriz Martínez[1,6,*], Filipa Simão[2,6], Verónica Gomes[3], Masinda Nguidi[2], Antonio Amorim[3,4], Elizeu F. Carvalho[2], Javier Marrugo[1], Leonor Gusmão[2]

[1]Molecular Genetics Laboratory, Institute for Immunological Research, University of Cartagena, Cartagena, Colombia
[2]DNA Diagnostic Laboratory (LDD), State University of Rio de Janeiro (UERJ), Rio de Janeiro, Brazil
[3]I IPATIMUP/i3S - Instituto de Investigação e Inovação em Saúde, Universidade do Porto, 4200-135 Porto, Portugal
[4]Faculty of Sciences of the University of Porto (FCUP), Porto, Portugal

*bmartineza1@unicartagena.edu.co

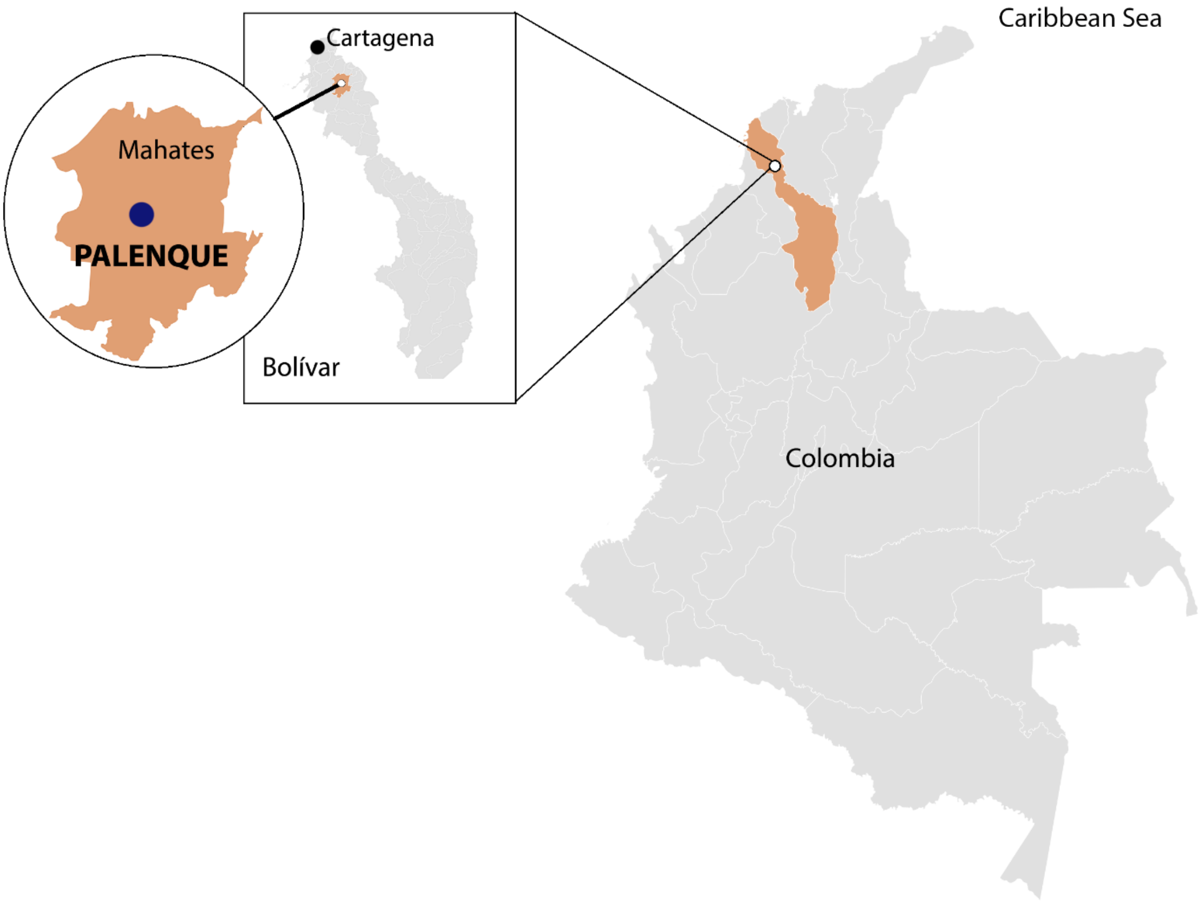

Supplementary Figure S1. Map of Colombia with the location of San Basilio de Palenque. The map was created using Big Maps Function on Excel v.2010 software.

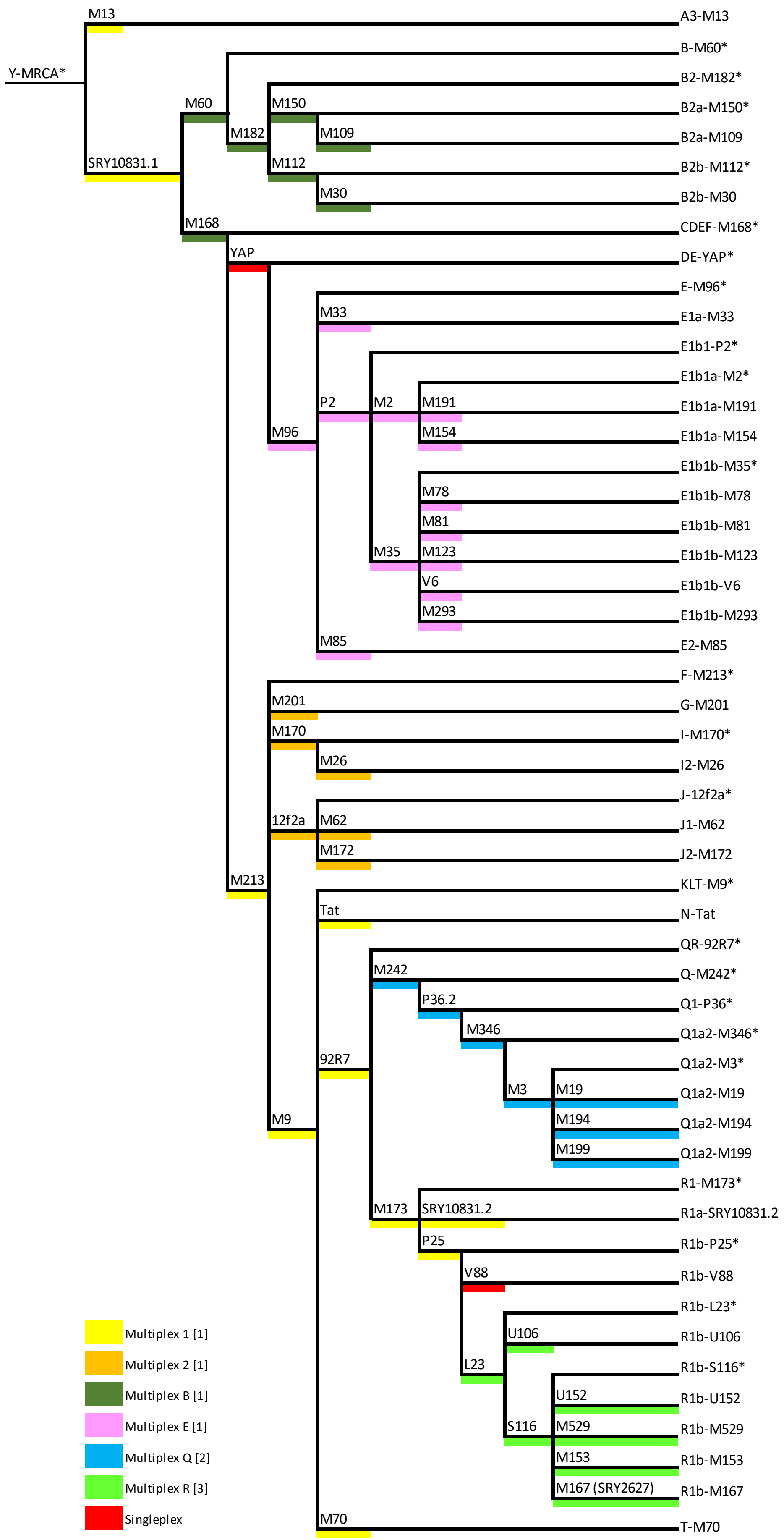

Supplementary Figure S2. Tree of Y-SNPs analysed in this study and methods used. Haplogroups are named in accordance with Van Oven et al. (2014) Hum Mutat 35:187-191. The Y-SNPs were chosen to discriminate the main African haplogroups that were expected to be found in San Basilio de Palenque, as well as those resulting from European and Native American admixture. [1] Gomes et al. (2010) *Hum Genet* 127:603-613; [2] Noguera et al. (2014) *Ann Hum Biol*. 41:453-459; [3] Resque et al. (2016) *PLoS One* 11:e0152573.

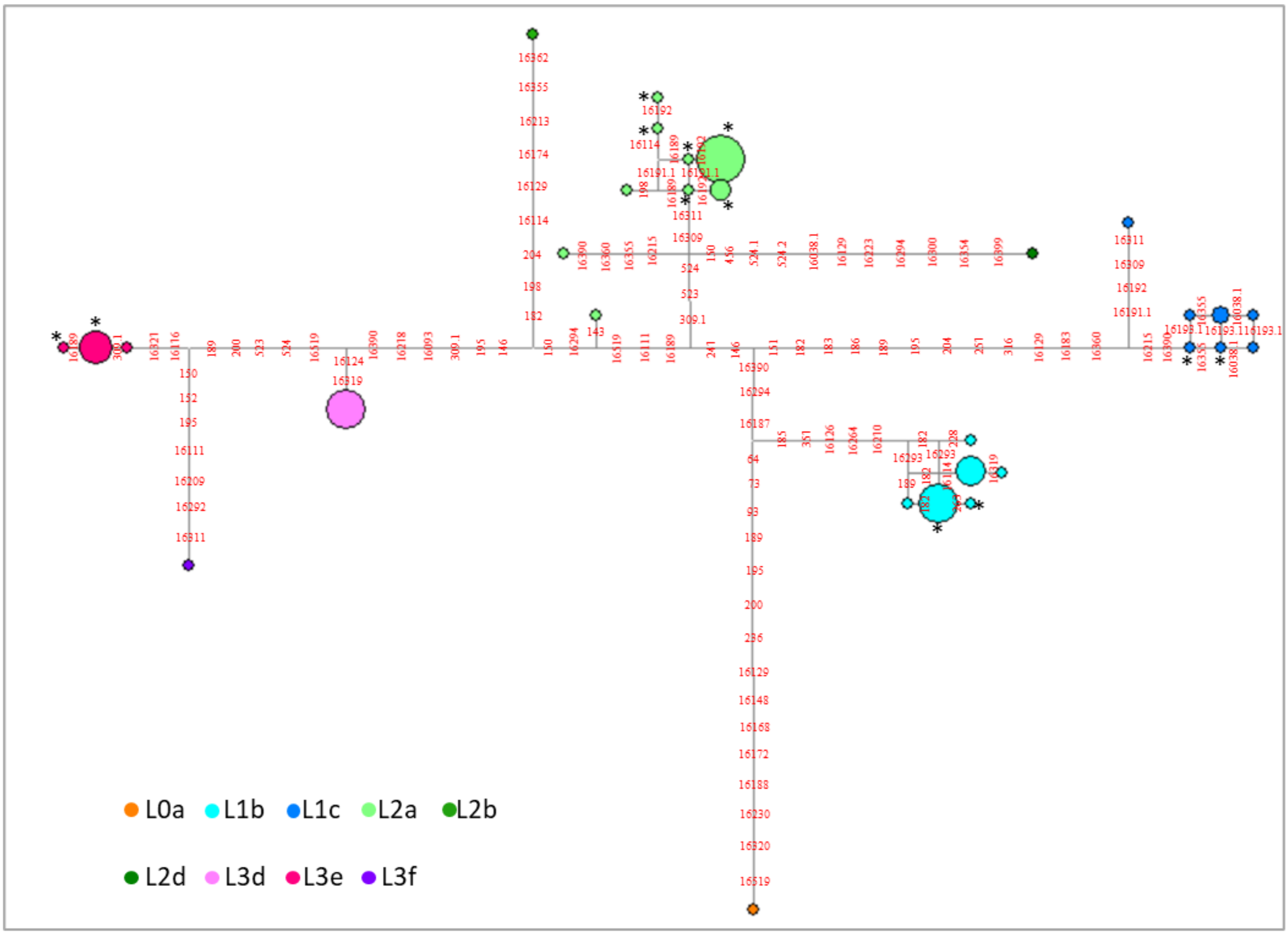

Supplementary Figure S3. Network representation of the full CR haplotypes (including indel polymorphisms) inside African haplogroups. The seven samples belonging to Native American haplogroups, representing 5 different haplogroups/haplotypes, were not included in the network. The polymorphic positions separating the haplotypes are described in red.

*haplotypes including one alternative sequences from samples with heteroplasmic position. The two possible variants for PR024, PR052, PR081, PU055, PU063 and PU103 (see haplotypes in table S2) were included in the network.

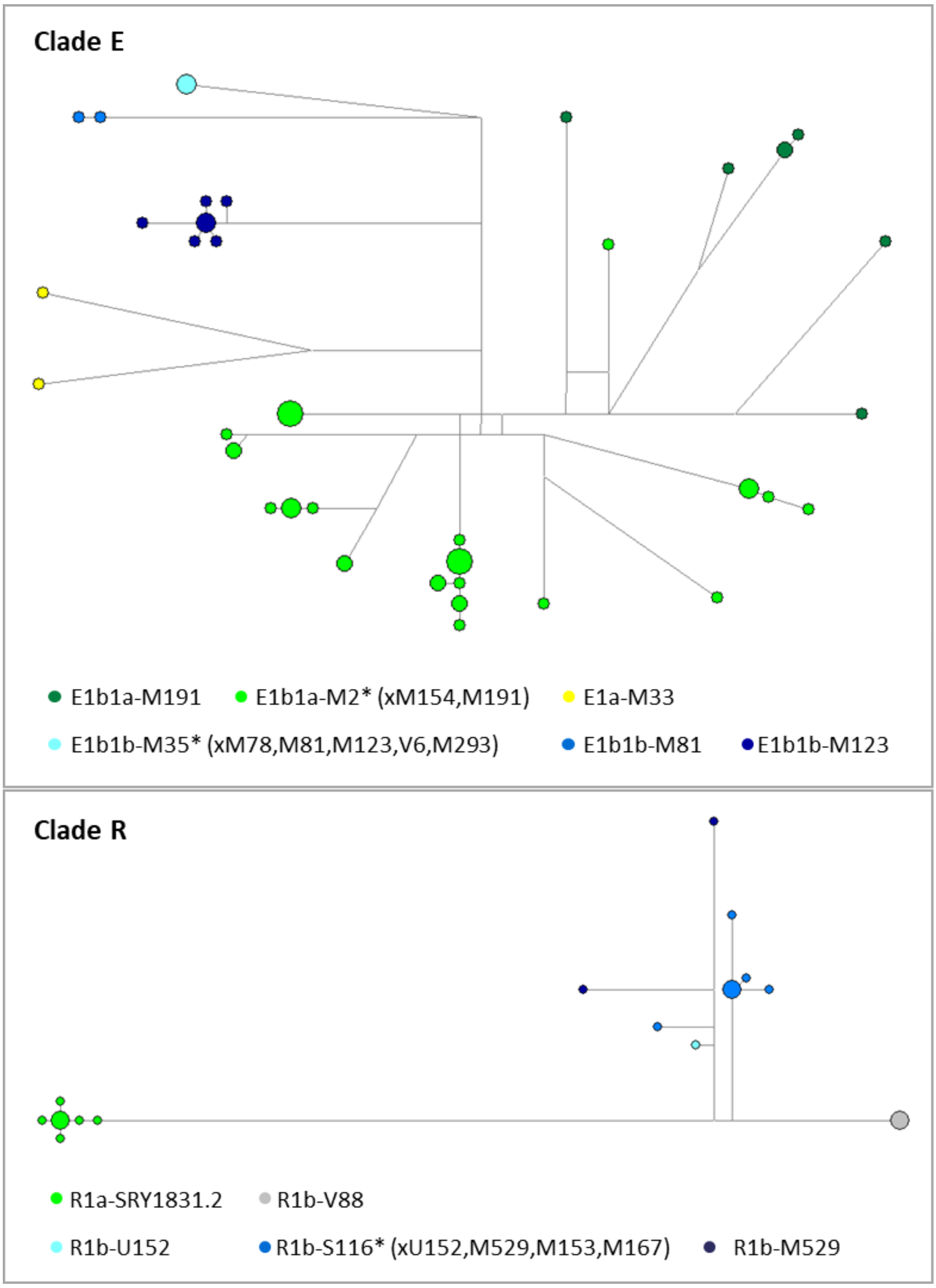

Supplementary Figure S4. Network representation of the 27 Y-STR based haplotypes found in Palenque inside clades E and R. Apart from the represented clades, the following haplogroups were also detected: B2a-M150* (xM109), with three samples sharing a single haplotype; G-M201, I2-M26 and J2-M172, one sample from each haplogroup; Q1a2-M3* (xM19,M194,M199), three samples only differing at DYF387S1; and Y-MRCA* (xM13,SRY10831.1), two samples only differing at DYF387S1.

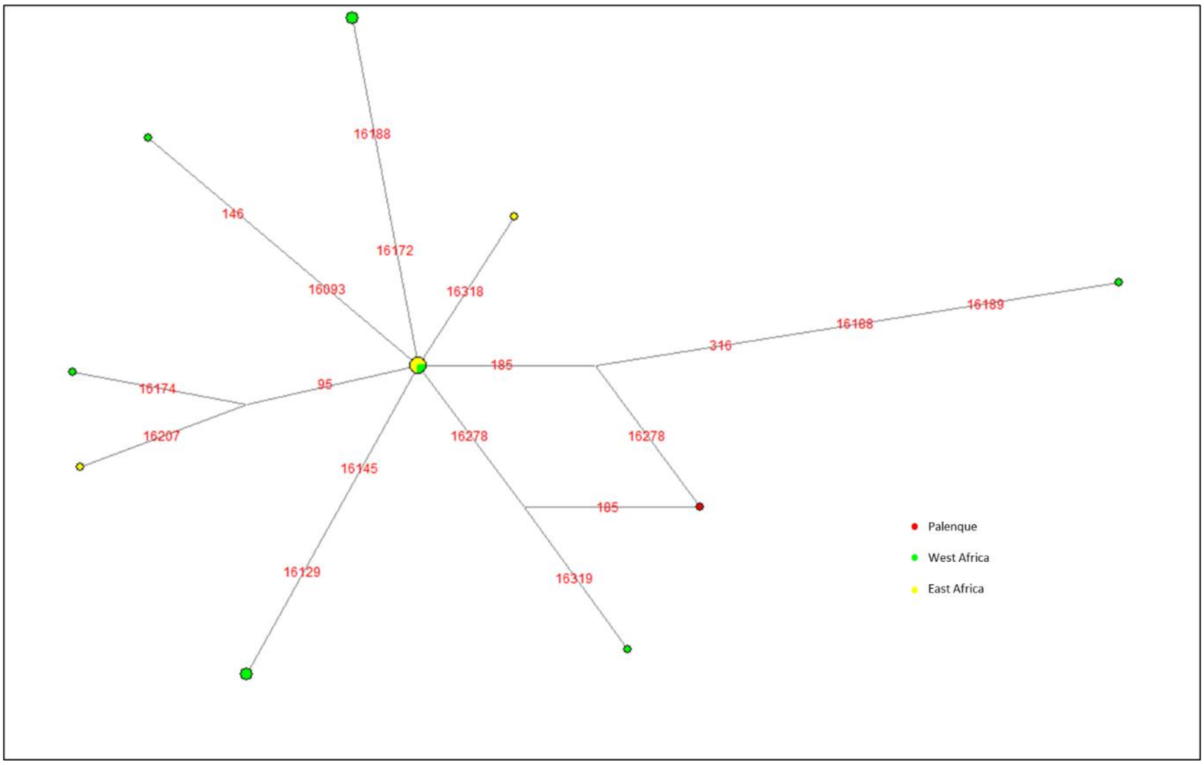

Supplementary Figure S5. Network representation of haplogroup L0a1a+200. The network was built using median-joining method [Bandelt et al. (1999). Mol Bio Evol. 16:37-48]. All haplotypes were framed between positions 16024-16365 (HVSI) and 73-340 (HVSII) and indels in positions 16193.xC, 309.xC and 315.xC were not considered. In samples with heteroplasmic samples, all variants were used. The polymorphic positions separating the haplotypes are described in red. The following samples were included: Palenque (n=1), Guinea Bissau (n=5), Kenya (n=1), Nigeria (n=1), Senegal (n=2), Togo (n=1) and Uganda (n=4). The samples were selected from the publications included in Supplementary Table S4.

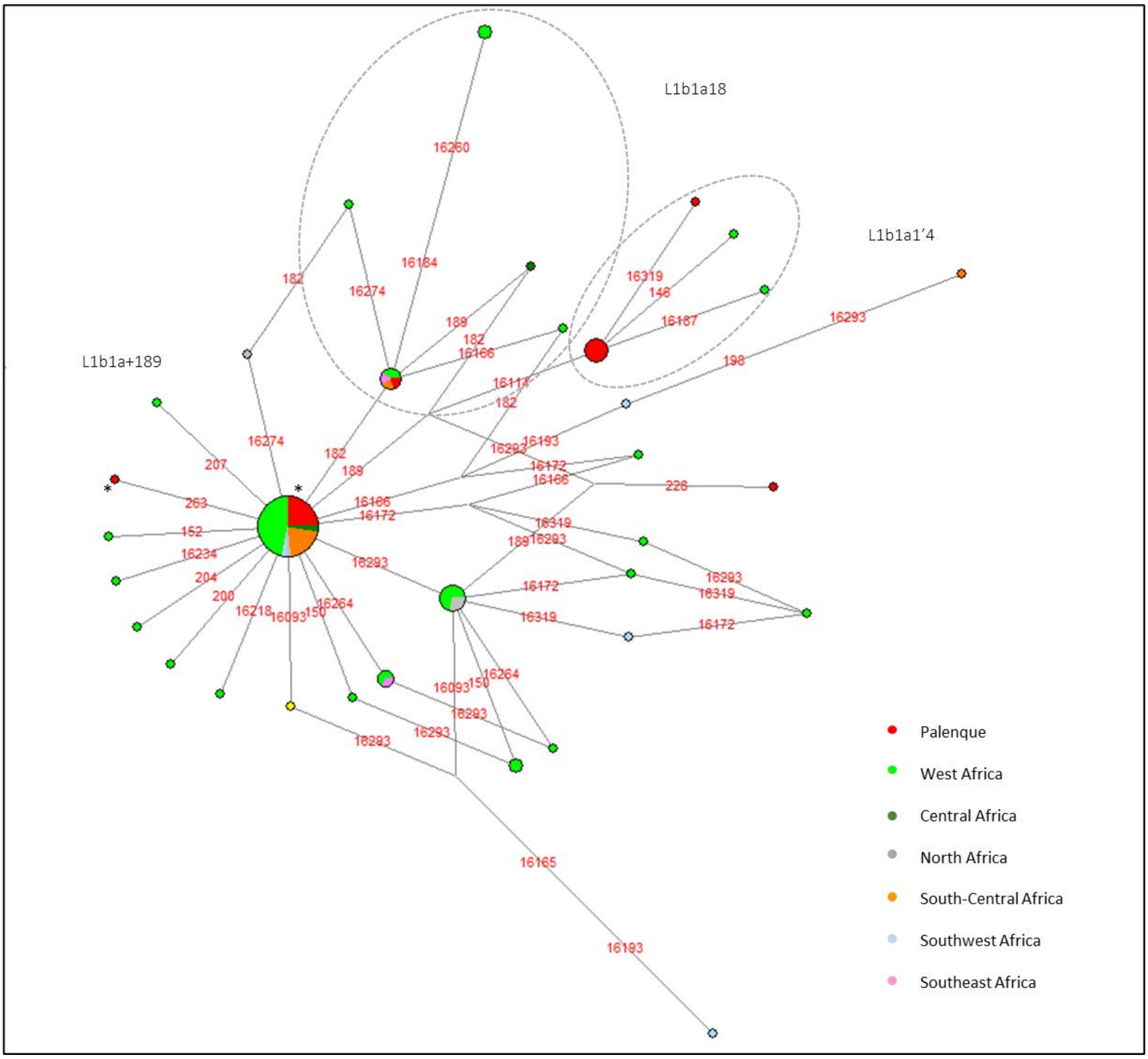

Supplementary Figure S6. Network representation of haplogroups inside L1b. The network was built using median-joining method [Bandelt et al. (1999). Mol Bio Evol. 16:37-48]. All haplotypes were framed between positions 16024-16365 (HVSI) and 73-340 (HVSII). Indels in positions 16193.xC, 309.xC and 315.xC were not considered. Both variants of heteroplasmic positions were considered. The polymorphic positions separating the haplotypes are described in red. The following samples were included: Palenque (n=19), Angola (n=5), Cameron (n=2), Chad (n=4), Ghana (n=9), Guinea Bissau (=1), Ivory Coast (n=4) , Kenya (n=1), Mali (n=1), Morocco (n=4), Mozambique (n=2), Niger (n=2), Nigeria (n=22), Senegal (n=2), Togo (n=2) and Zambia (n=10). The samples were selected from the publications included in Supplementary Table S4.

*haplotypes including one alternative sequences from samples with heteroplasmic position. The two possible variants for PU055 (see haplotype in table S2) were included in the network.

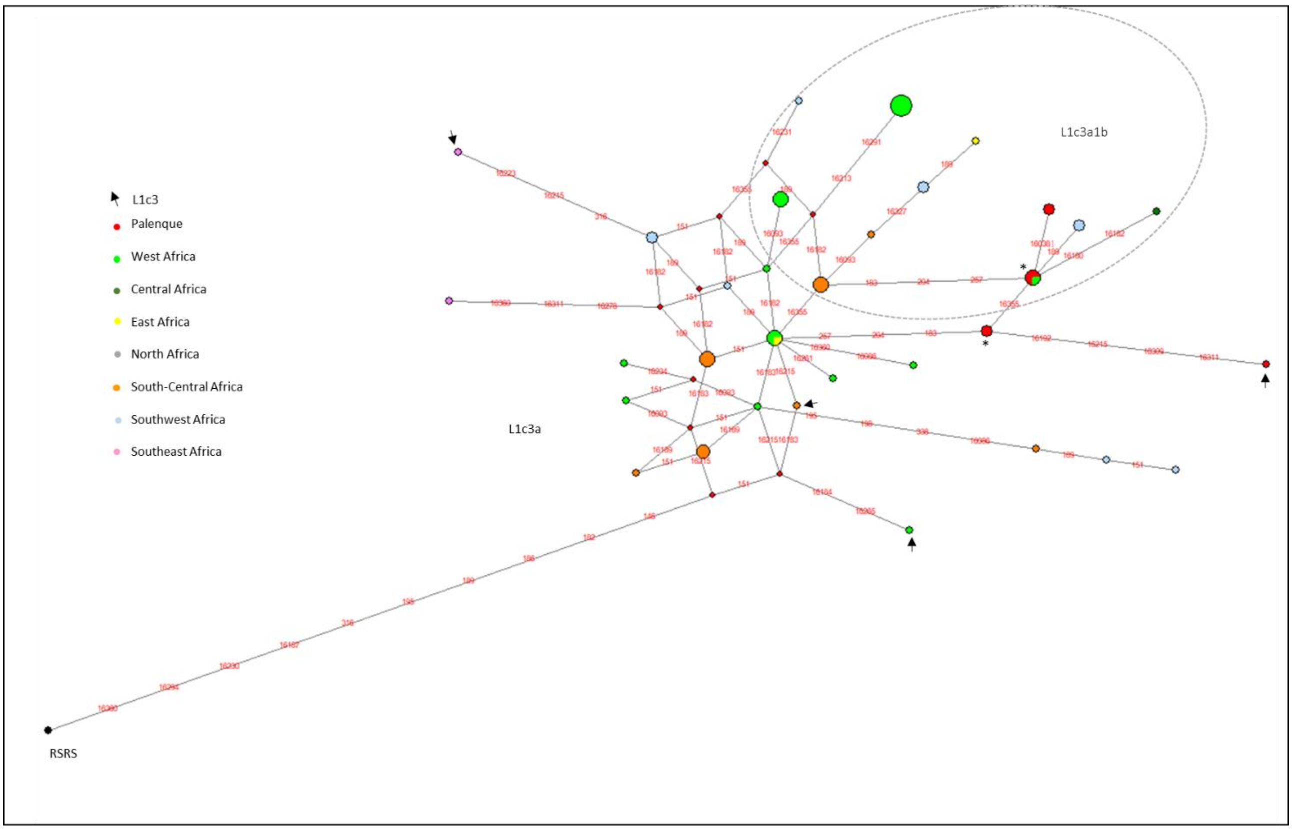

Supplementary Figure S7. Network representation of haplogroups inside L1c. The network was built using median-joining method [Bandelt et al. (1999). Mol Bio Evol. 16:37-48]. All haplotypes were framed between positions 16024-16365 (HVSI) and 73-340 (HVSII) and indels in positions 16193.xC, 309.xC and 315.xC were not considered. Both variants of heteroplasmic positions were considered. The polymorphic positions separating the haplotypes are described in red. The following samples were included: Palenque (n=7), Angola (n=10), Cameron (n=1), Gambia (n=1), Ghana (n=16), Ivory Coast (n=3), Mozambique (n=2), Niger (n=1), Nigeria (n=1), Rwanda (n=1), Uganda (n=1) and Zambia (n=15). The samples were selected from the publications included in Supplementary Table S4.

*haplotypes including one alternative sequences from samples with heteroplasmic position. The two possible variants for PU103 (see haplotype in table S2) were included in the network.

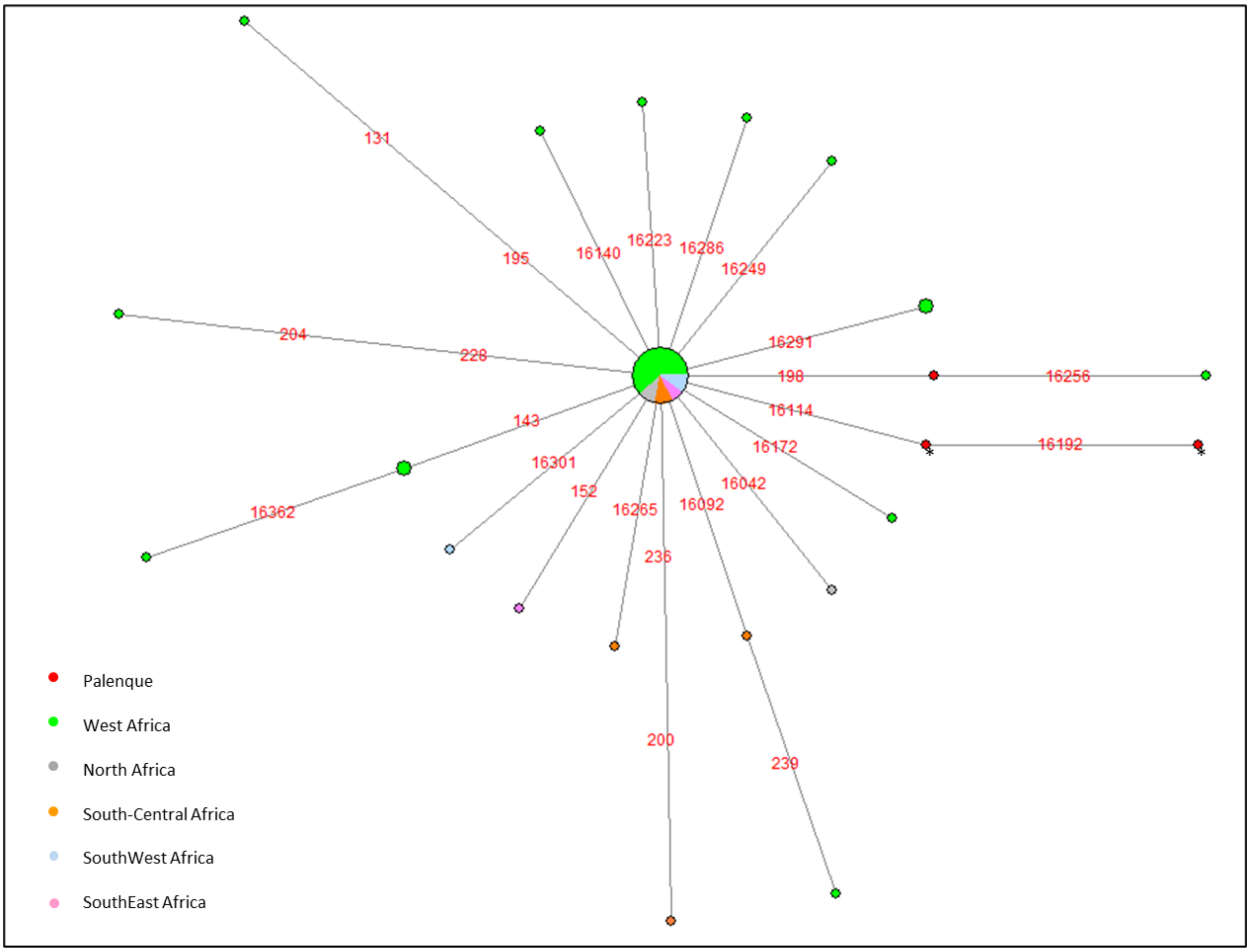

Supplementary Figure S8. Network representation of haplogroup L2a1. The network was built using median-joining method [Bandelt et al. (1999). Mol Bio Evol. 16:37-48]. All haplotypes were framed between positions 16024-16365 (HVSI) and 73-340 (HVSII) and indels in positions 16193.xC, 309.xC and 315.xC were not considered. Both variants of heteroplasmic positions were considered. The polymorphic positions separating the haplotypes are described in red. The following samples were included: Palenque (n=2), Angola (n=4), Burkina Faso (n=1), Chad (n=1), Ghana (n=11), Guinea Bissau (n=3), Ivory Coast (n=6), Mali (n=1), Morocco (n=3), Mozambique (n=3), Nigeria (n=6), Togo (n=2), Tunisia (n=1) and Zambia (n=6). The samples were selected from the publications included in Supplementary Table S4.

*haplotypes including one alternative sequences from samples with heteroplasmic position. The two possible variants for PU063 (see haplotype in table S2) were included in the network.

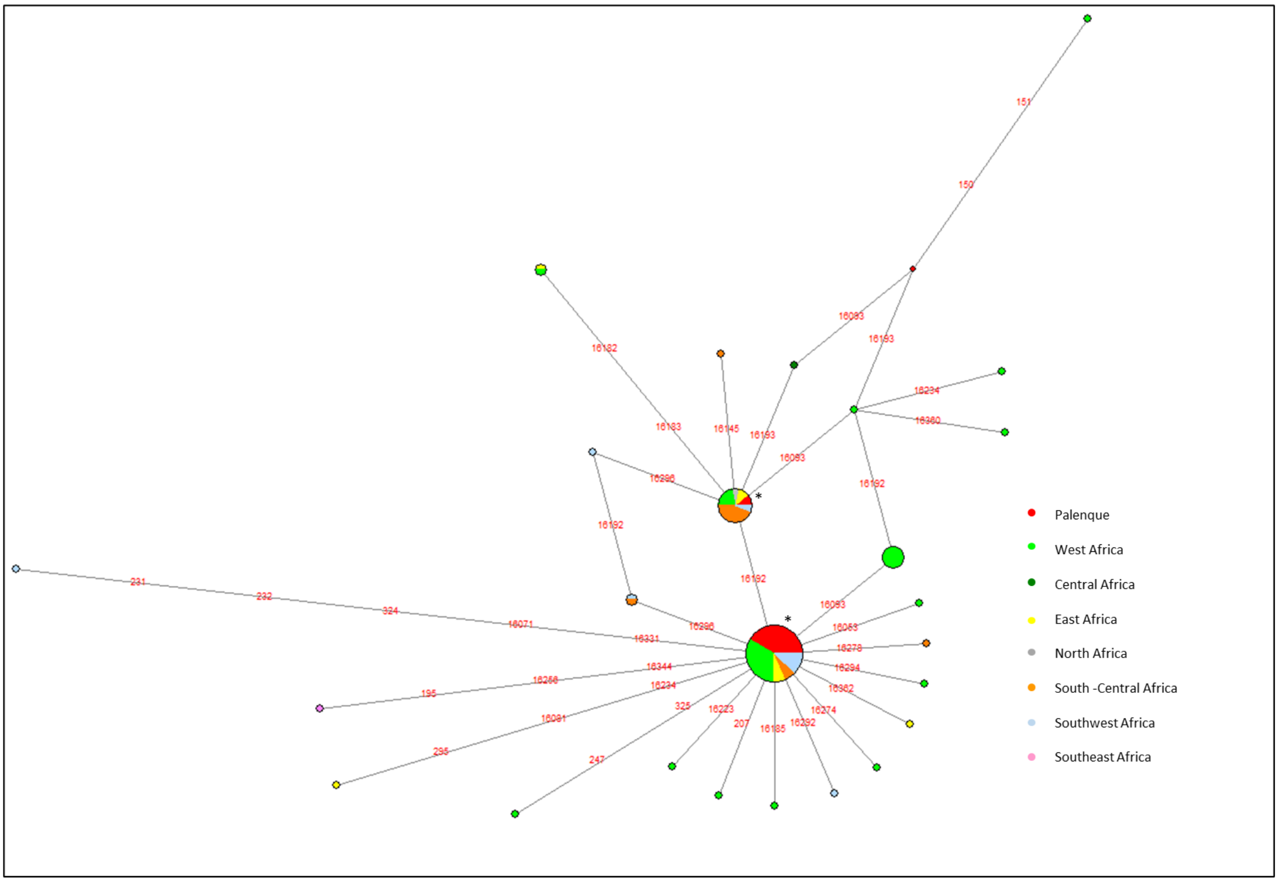

Supplementary Figure S9. Network representation of haplogroup L2a1+16189(16192). The network was built using median-joining method [Bandelt et al. (1999). Mol Bio Evol. 16:37-48]. All haplotypes were framed between positions 16024-16365 (HVSI) and 73-340 (HVSII) and indels in positions 16193.xC, 309.xC and 315.xC were not considered. Both variants of heteroplasmic positions were considered. The polymorphic positions separating the haplotypes are described in red. The following samples were included: Palenque (n=20), Angola (n=12), Benin (n=1), Cameroon (n=1), Gambia (n=1), Ghana (n=14), Guinea Bissau (n=1), Ivory Coast (n=4), Kenya (n=2), Mozambique (n=1), Niger (n=6), Nigeria (n=11), Togo (n=1), Tunisia (n=1), Uganda (n=5) and Zambia (n=15). The samples were selected from the publications included in Supplementary Table S4.

*haplotypes including one alternative sequences from samples with heteroplasmic position. The two possible variants for PR024 and PR081 (see haplotypes in table S2) were included in the network.

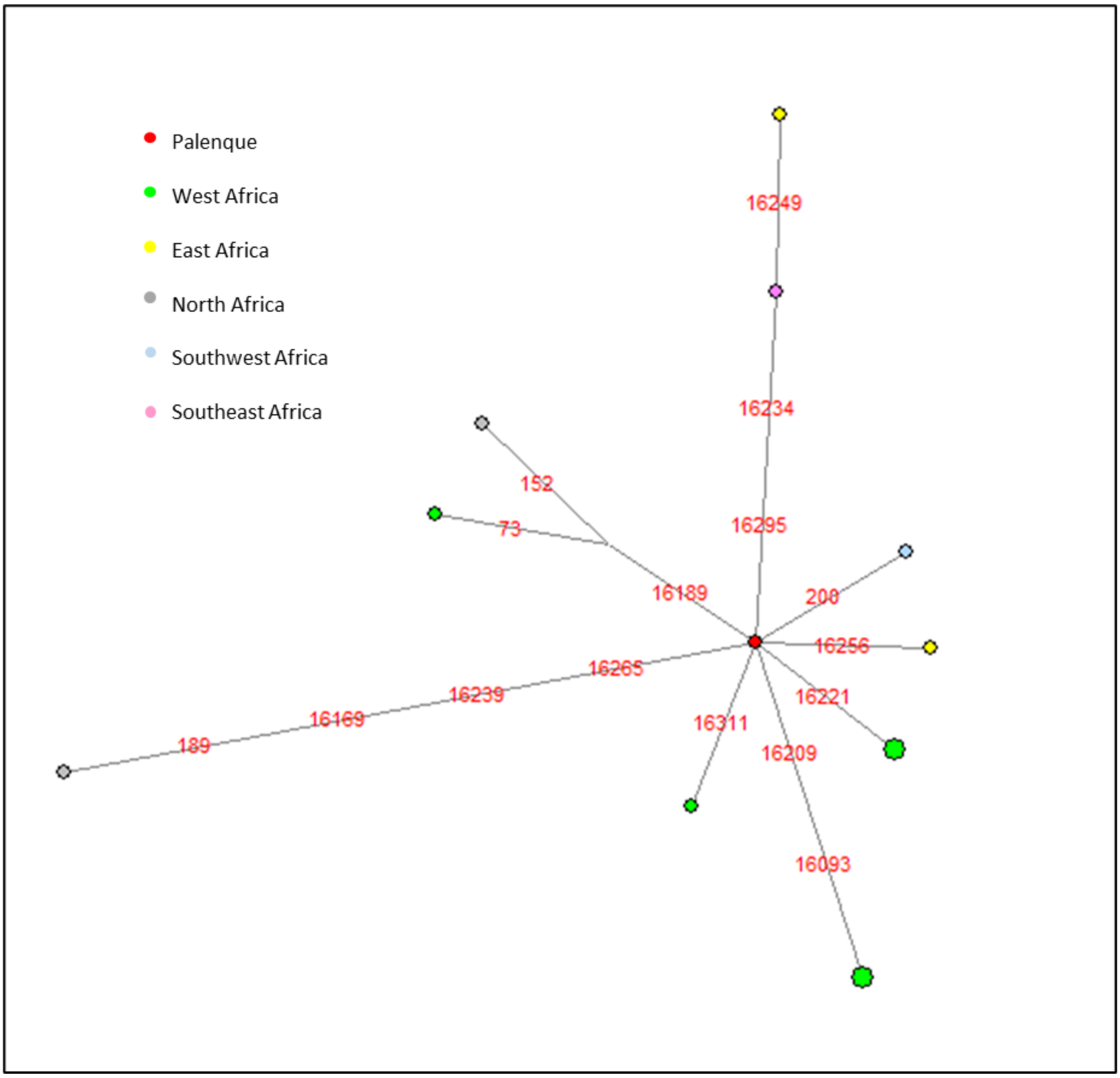

Supplementary Figure S10. Network representation of haplogroup L2a1+143+16309. The network was built using median-joining method [Bandelt et al. (1999). Mol Bio Evol. 16:37-48]. All haplotypes were framed between positions 16024-16365 (HVSI) and 73-340 (HVSII) and indels in positions 16193.xC, 309.xC and 315.xC were not considered. Both variants of heteroplasmic positions were considered. The polymorphic positions separating the haplotypes are described in red. The following samples were included: Palenque (n=1), Angola (n=1), Burkina Faso (n=1), Chad (n=2), Ghana (n=2), Kenya (n=1), Morocco (n=2), Mozambique (n=1), Nigeria (n=1) and Uganda (n=1). The samples were selected from the publications included in Supplementary Table S4.

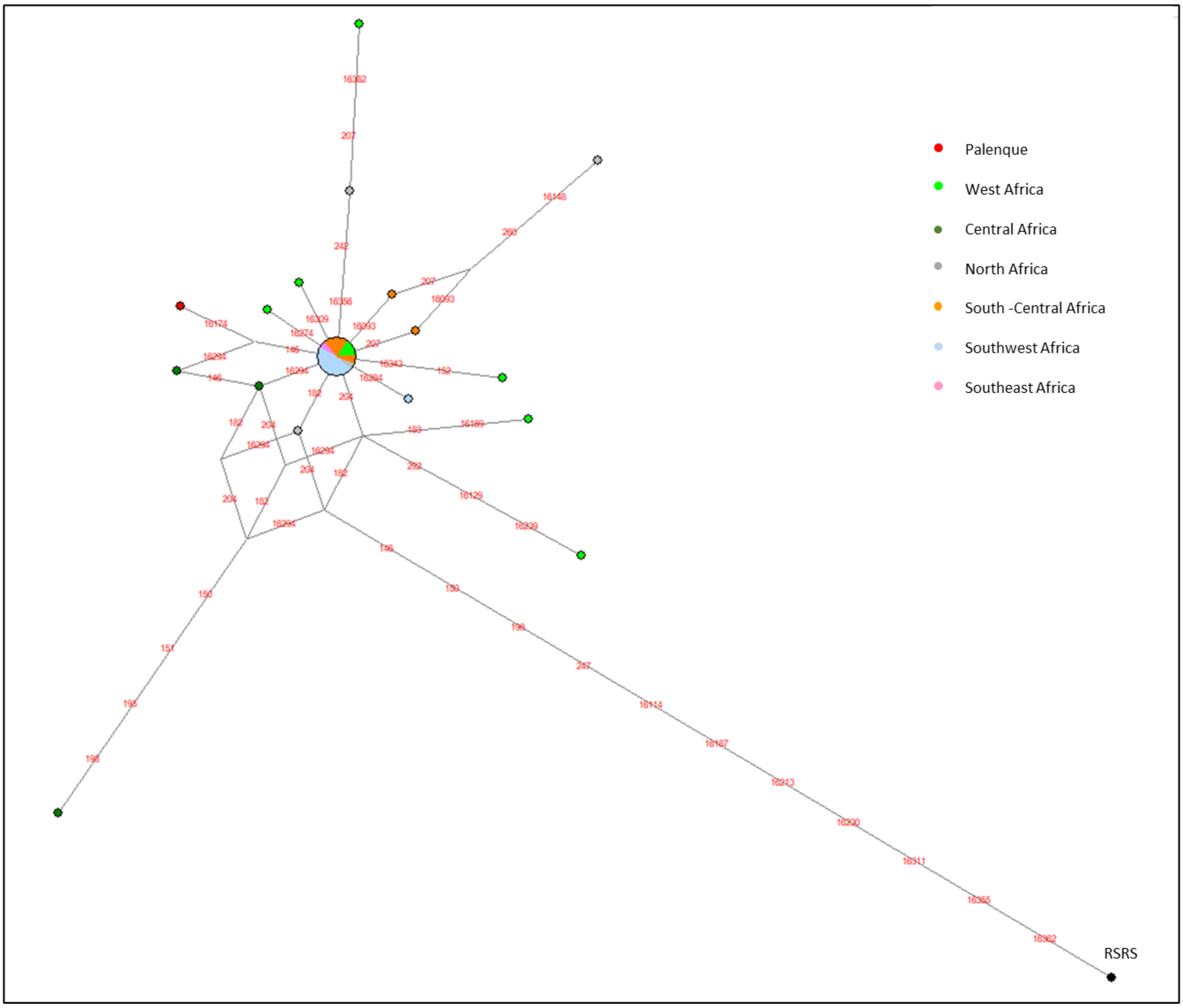

Supplementary Figure S11. Network representation of haplogroup L2b1a. The network was built using median-joining method [Bandelt et al. (1999). Mol Bio Evol. 16:37-48]. All haplotypes were framed between positions 16024-16365 (HVSI) and 73-340 (HVSII) and indels in positions 16193.xC, 309.xC and 315.xC were not considered. Both variants of heteroplasmic positions were considered. The polymorphic positions separating the haplotypes are described in red. The following samples were included: Palenque (n=1), Angola (n=10), Cameroon (n=3), Chana (n=1), Guinea Bissau (n=1), Ivory Coast (n=1), Morocco (n=4), Mozambique (n=1), Nigeria (n=5), Senegal (n=1) and Zambia (n=7). The samples were selected from the publications included in Supplementary Table S4.

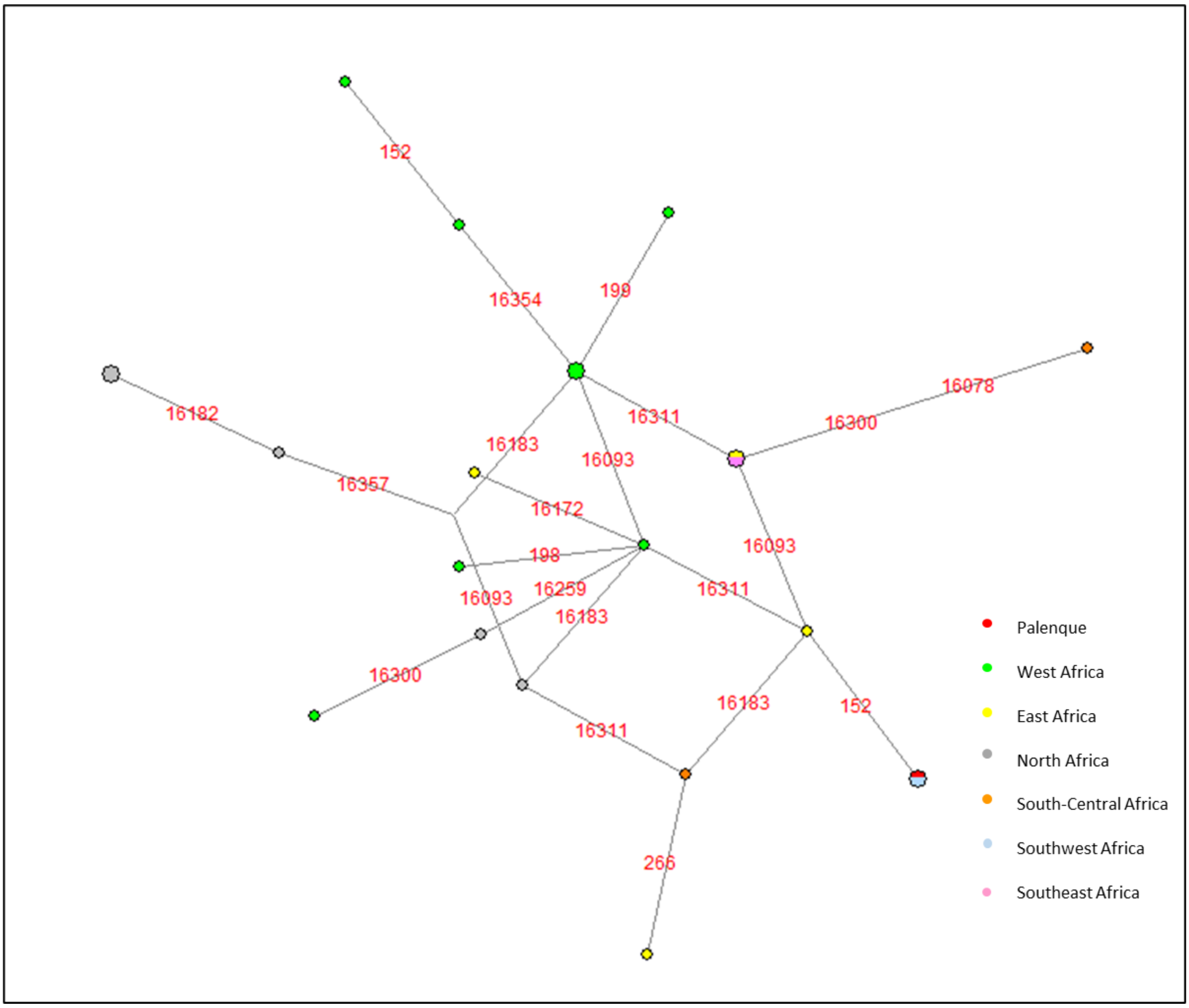

Supplementary Figure S12. Network representation of haplogroup L2d+16129. The network was built using median-joining method [Bandelt et al. (1999). Mol Bio Evol. 16:37-48]. All haplotypes were framed between positions 16024-16365 (HVSI) and 73-340 (HVSII) and indels in positions 16193.xC, 309.xC and 315.xC were not considered. Both variants of heteroplasmic positions were considered. The polymorphic positions separating the haplotypes are described in red. The following samples were included: Palenque (n=1), Angola (n=1), Guinea Bissau (n=2), Ivory Coast (n=4), Kenya (n=1), Morocco (n=5), Mozambique (n=1), Nigeria (n=2), Rwanda (n=3) and Zambia (n=2). The samples were selected from the publications included in Supplementary Table S4.

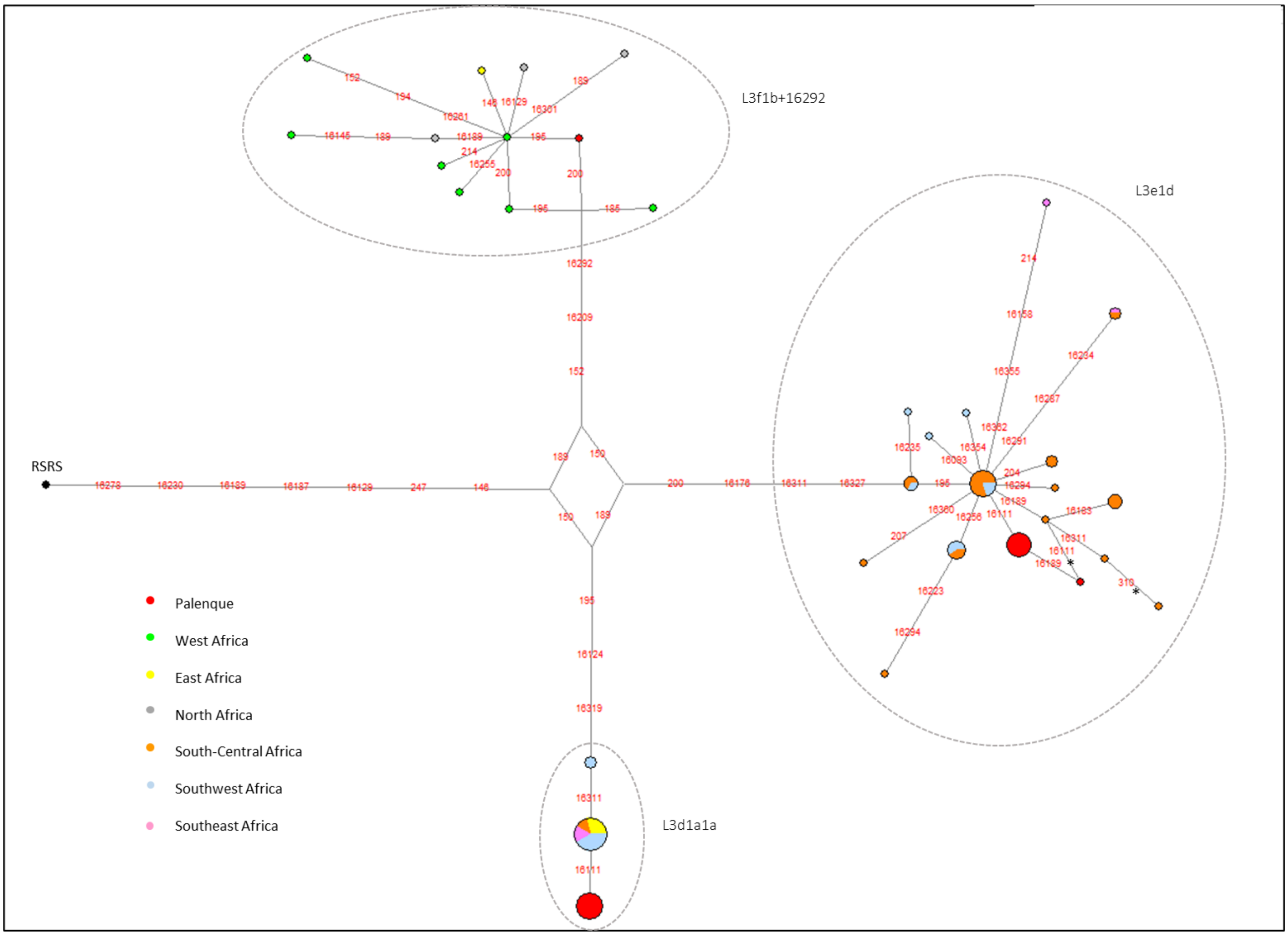

Supplementary Figure S13. Network representation of haplogroups inside L3. The network was built using median-joining method [Bandelt et al. (1999). Mol Bio Evol. 16:37-48]. All haplotypes were framed between positions 16024-16365 (HVSI) and 73-340 (HVSII) and indels in positions 16193.xC, 309.xC and 315.xC were not considered. The polymorphic positions separating the haplotypes are described in red. The following samples were included: Palenque (n=20), Angola (n=18), Chad (n=3), Ghana (n=2), Kenya (n=1), Morocco (n=3), Mozambique (n=5), Niger (n=2), Rwanda (n=2), Uganda (n=3) and Zambia (n=26). The samples were selected from the publications included in Supplementary Table S4.

*haplotypes including one alternative sequences from samples with heteroplasmic position. The two possible variants for PR052 (see haplotype in table S2) were included in the network.

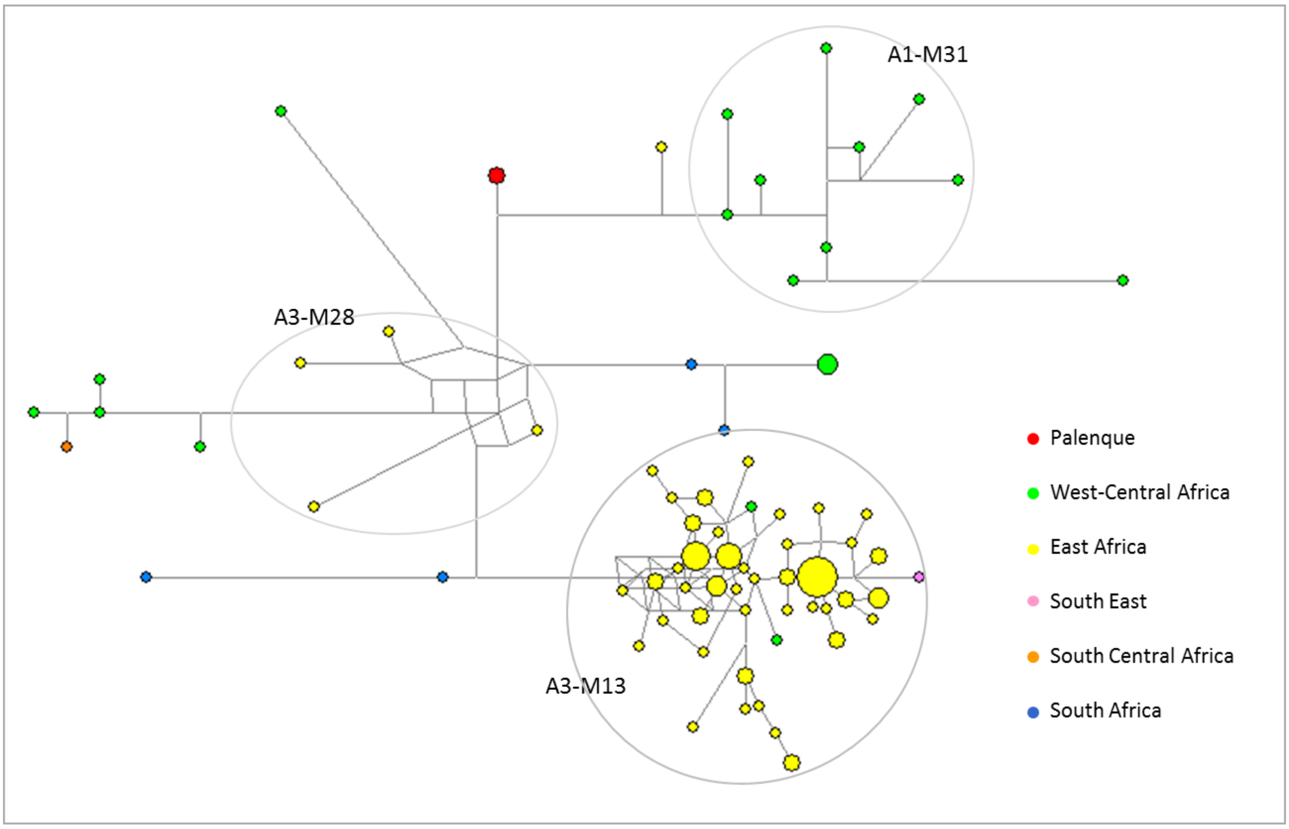

Supplementary Figure S14. Network representation of haplotypes selected that could belong to Y-MRCA* (xM13,SRY10831.1). The network was built using 12 Y-STR loci and resolved by applying reduced median [Bandelt et al. (1995). Genetics. 141:743-753] and median-joining [Bandelt et al. (1999). Mol Bio Evol. 16:37-48] methods sequentially. The following samples were included: Palenque (n=2) Benin (n=1), Botswana (n=1), Burkina Faso (n=1), Equatorial Guinea (n=1), Eritrea (n=9), Ethiopia (n=22), Gabon (n=4), Gabon/Cameroon Pygmy (n=3), Guinea Bissau (n=8), Ivory Coast (n=1), Kenya (n=11), Mozambique (n=1), Namibia (n=3), Nigeria (n=1), Uganda (n=39), Zambia (n=1). The samples were selected from the publications included in Supplementary Table S5.

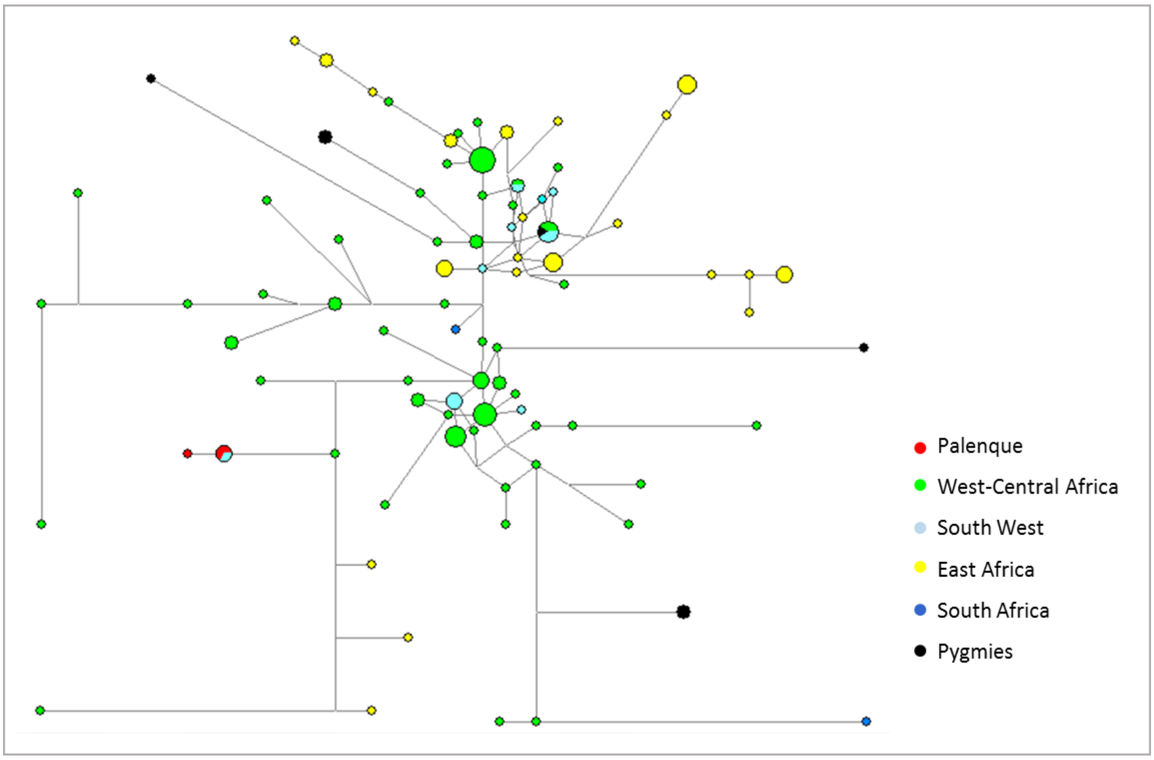

Supplementary Figure S15. Network representation of haplotypes selected inside clade B-M60 (except those known to carrying the M109/M152 derived allele). The number of Y-STRs used to construct the network was reduced to a common set of 11 loci: DYS389I, DYS389II, DYS19, DYS390, DYS438, DYS392, DYS437, DYS385a/b, DYS393, and DYS439. The network was resolved by applying reduced median [Bandelt et al. (1995). Genetics. 141:743-753] and median-joining [Bandelt et al. (1999). Mol Bio Evol. 16:37-48] methods sequentially. The following 134 African samples were included: Palenque (n=3), Angola (n=12), Benin (n=4), Burkina Faso (n=7), Cameroon (n=9), Gabon/Cameroon Pygmy (n=6), DRC (n=1), Eritrea (n=3), Gabon (n=48), Ghana (n=1), Guinea Bissau (n=1), Namibia (n=1), Niger (n=11), Nigeria (n=4), South Africa (n=1) and Uganda (n=25). The samples were selected from the publications included in Supplementary Table S5.

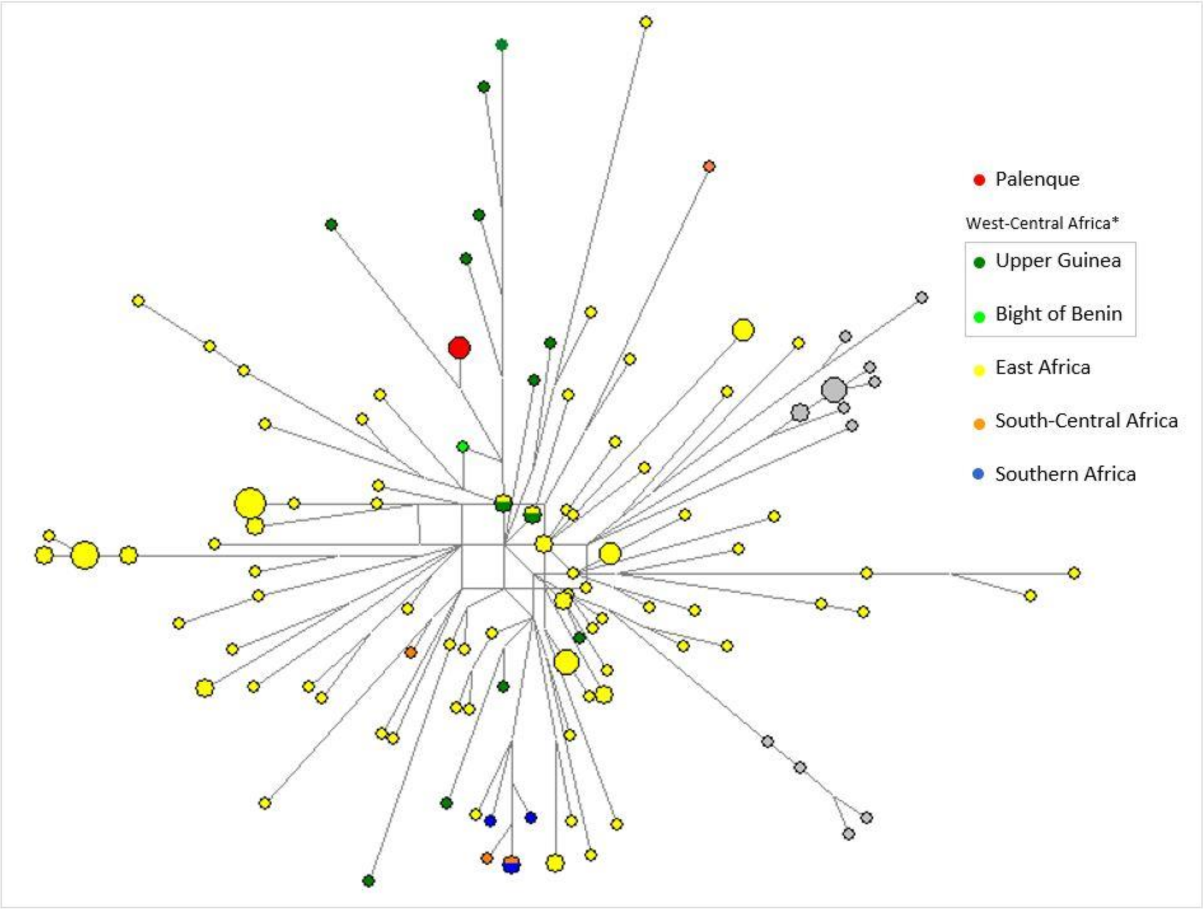

Supplementary Figure S16. Network representation of haplotypes selected inside clade E1b1b-M35 (except those carrying the M78, M81, M123, V6, M293 derived alleles). The network was built using available information on 142 African samples from haplogroup E1b1b-M35 (those carrying the M78, M81, M123, V6, M293 derived allele were not included). The number of Y-STRs used to construct the network was reduced to a common set of 12 loci: DYS389I, DYS389II, DYS19, DYS390, DYS391, DYS438, DYS392, DYS437, DYS385a/b, DYS393, and DYS439. The network was resolved by applying reduced median [Bandelt et al. (1995). Genetics. 141:743-753] and median-joining [Bandelt et al. (1999). Mol Bio Evol. 16:37-48] methods sequentially. The following samples were included: Palenque (n=3), Algeria (n=16), Benin (n=1), Botswana (n=2), Burkina Faso (n=7), Eritrea (n=23), Ethiopia (n=45), Guinea Bissau (n=6), Kenya (n=24), Senegal (n=1), South Africa (n=1), Uganda (n=7) and Zambia (n=4). The samples were selected from the publications included in Supplementary Table S5.

*Samples from West-Central region were discriminated between Upper Guinea and Bight of Benin, for a clearer inference of the origin of the samples from Palenque.

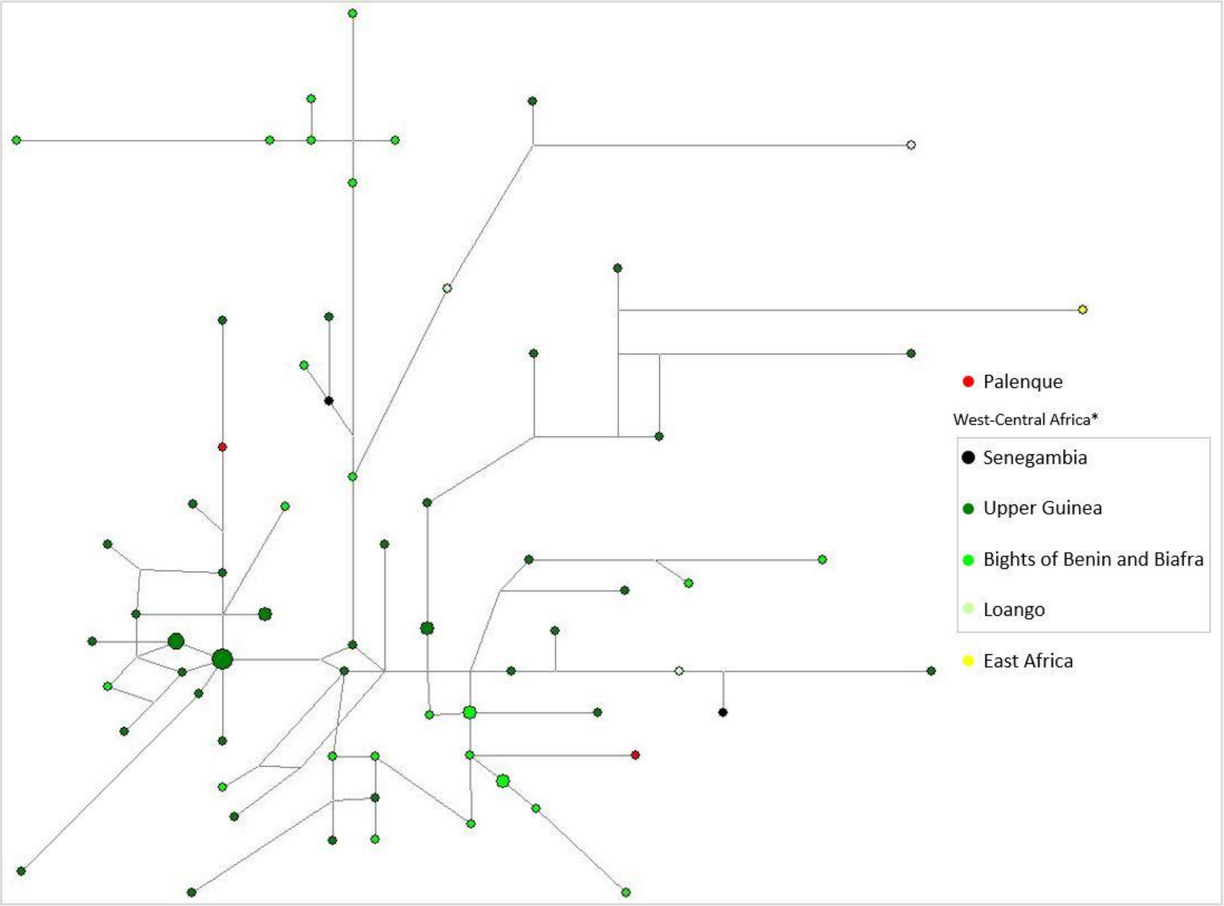

Supplementary Figure S17. Network representation of haplotypes selected inside from haplogroup E1a-M33. The network was built using 11 Y-STR loci and resolved by applying reduced median [Bandelt et al. (1995). Genetics. 141:743-753] and median-joining [Bandelt et al. (1999). Mol Bio Evol. 16:37-48] methods sequentially. The following samples were included: Palenque (n=4), Benin (n=7), Burkina Faso (n=26), Equatorial Guinea (n=23), Ethiopia (n=1), Gabon (n=3), Guinea Bissau (n=17), Ivory Coast (n=1), Nigeria (n=7), Senegal (n=2) and Togo (n=2). The samples were selected from the publications included in Supplementary Table S5.

*Samples from West-Central region were discriminated between Senegambia, Upper Guinea, Bights of Benin and Biafra, and Loango, for a clearer inference of the origin of the samples from Palenque.

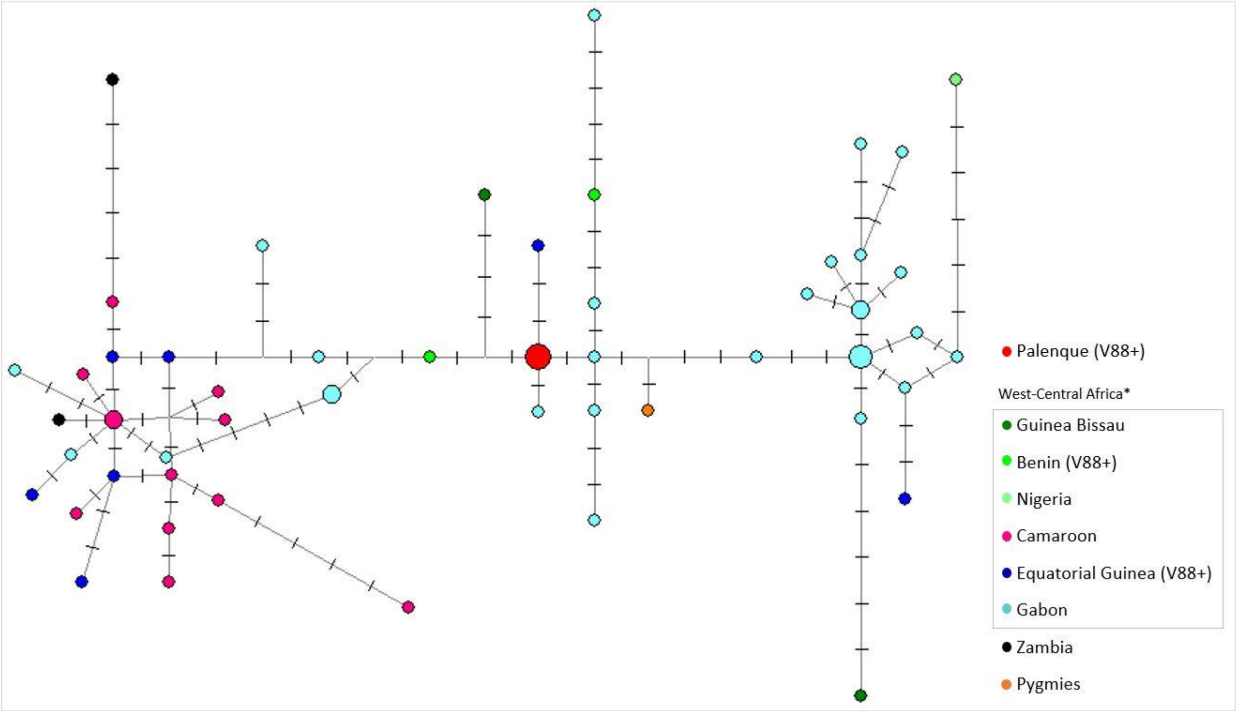

Supplementary Figure S18. Network representation of haplotypes selected inside clade R1b-P25 (excluding those assigned to sub-lineages outside the R1b-V88 branch). The network was built by applying reduced median [Bandelt et al. (1995). Genetics. 141:743-753] and median-joining [Bandelt et al. (1999). Mol Bio Evol. 16:37-48] methods sequentially. The samples were selected from the publications included in Supplementary Table S5. The selected samples are mostly from western Africa, except two samples from Pygmies and one from Zambia. Two samples from Benin and 7 from Equatorial Guinea have the V88 derived allele. The remaining samples were not typed for the SNP V88, although they belong to higher clades that may include this mutation. The number of Y-STRs used to construct the network was reduced to a common set of 11 loci: DYS389I, DYS389II, DYS19, DYS390, DYS438, DYS392, DYS437, DYS385a/b, DYS393, and DYS439. The presence of an intermediate allele at DYS385 was also considered in the construction of the network, by coding it as a biallelic marker.

*Samples from West-Central region were discriminated by country, for a clearer inference of the origin of the samples from Palenque.
